# Impact of *ADCY9* Genotype on Response to Anacetrapib

**DOI:** 10.1161/CIRCULATIONAHA.119.041546

**Published:** 2019-07-23

**Authors:** Jemma C. Hopewell, Maysson Ibrahim, Michael Hill, Peter M. Shaw, Eugene Braunwald, Robert O. Blaustein, Louise Bowman, Martin J. Landray, Marc S. Sabatine, Rory Collins

**Affiliations:** 1 Clinical Trial Service Unit and Epidemiological Studies Unit (J.C.H., M.I., M.H., L.B., M.J.L., R.C.), Nuffield Department of Population Health, University of Oxford, United Kingdom.; 2 Medical Research Council Population Health Research Unit (M.H., L.B.), Nuffield Department of Population Health, University of Oxford, United Kingdom.; 3 Department of Genetics and Pharmacogenomics (P.M.S.), Merck Research Laboratories, Merck & Co., Inc., Kenilworth, NJ.; 4 Cardiovascular Clinical Research (R.O.B.), Merck Research Laboratories, Merck & Co., Inc., Kenilworth, NJ.; 5 TIMI Study Group, Division of Cardiovascular Medicine, Brigham and Women’s Hospital and Harvard Medical School, Boston, MA (E.B., M.S.S.).

**Keywords:** adenylate cyclase 9, anacetrapib, cholesterol ester transfer protein, pharmacogenetics, randomized controlled trial

## Abstract

Exploratory analyses of previous randomized trials generated a hypothesis that the clinical response to cholesteryl ester transfer protein (CETP) inhibitor therapy differs by *ADCY9* genotype, prompting the ongoing dal-GenE trial in individuals with a particular genetic profile. The randomized placebo-controlled REVEAL trial (Randomized Evaluation of the Effects of Anacetrapib through Lipid-Modification) demonstrated the clinical efficacy of the CETP inhibitor anacetrapib among patients with preexisting atherosclerotic vascular disease. In the present study, we examined the impact of *ADCY9* genotype on response to anacetrapib in the REVEAL trial.

**Methods:**

Individuals with stable atherosclerotic vascular disease who were treated with intensive atorvastatin therapy received either anacetrapib 100 mg daily or matching placebo. Cox proportional hazards models, adjusted for the first 5 principal components of ancestry, were used to estimate the effects of allocation to anacetrapib on major vascular events (a composite of coronary death, myocardial infarction, coronary revascularization, or presumed ischemic stroke) and the interaction with *ADCY9* rs1967309 genotype.

**Results:**

Among 19 210 genotyped individuals of European ancestry, 2504 (13.0%) had a first major vascular event during 4 years median follow-up: 1216 (12.6%) among anacetrapib-allocated participants and 1288 (13.4%) among placebo-allocated participants. Proportional reductions in the risk of major vascular events with anacetrapib did not differ significantly by *ADCY9* genotype: hazard ratio (HR) = 0.92 (95% CI, 0.81–1.05) for GG; HR = 0.94 (95% CI, 0.84–1.06) for AG; and HR = 0.93 (95% CI, 0.76–1.13) for AA genotype carriers, respectively; genotypic *P* for interaction = 0.96. Furthermore, there were no associations between *ADCY9* genotype and the proportional reductions in the separate components of major vascular events or meaningful differences in lipid response to anacetrapib.

**Conclusions:**

The REVEAL trial is the single largest study to date evaluating the *ADCY9* pharmacogenetic interaction. It provides no support for the hypothesis that *ADCY9* genotype is materially relevant to the clinical effects of the CETP inhibitor anacetrapib. The ongoing dal-GenE study will provide direct evidence as to whether there is any specific pharmacogenetic interaction with dalcetrapib.

**Clinical Trial Registration:**

URL: https://www.clinicaltrials.gov. Unique identifier: NCT01252953. URL: http://www.isrctn.com. Unique identifier: ISRCTN48678192. URL: https://www.clinicaltrialsregister.eu. Unique identifier: 2010-023467-18.

## Abstract 2

Supplemental Digital Content is available in the text.

**Clinical Perspective**

**What Is New?**

- Exploratory analyses of previous randomized trials generated a hypothesis that clinical response to cholesteryl ester transfer protein (CETP) inhibitor therapy differs by *ADCY9* genotype.
- The REVEAL trial (Randomized Evaluation of the Effects of Anacetrapib through Lipid-Modification) is the single largest study to date evaluating the *ADCY9* pharmacogenetic interaction and has provided robust evidence that *ADCY9* genotype does not affect response to the CETP inhibitor anacetrapib.

**What Are the Clinical Implications?**

- The REVEAL trial indicated that the CETP inhibitor anacetrapib reduced the risk of major vascular events to the extent expected from the reduction in non–high-density lipoprotein cholesterol that was produced.
- The present analyses do not support the hypothesis that the effects of anacetrapib are materially altered by *ADCY9* genotype, although an effect that is specific to another CETP inhibitor cannot be ruled out.
- The ongoing dal-GenE trial includes patients on the basis of a specific *ADCY9* genotype and will directly assess its relevance to dalcetrapib.

Recently, the randomized placebo-controlled REVEAL trial (Randomized Evaluation of the Effects of Anacetrapib through Lipid-Modification) showed that a median 4 years of treatment with the cholesteryl ester transfer protein (CETP) inhibitor anacetrapib significantly reduced the incidence of cardiovascular outcomes among 30 449 patients with preexisting atherosclerotic vascular disease who were receiving effective low-density lipoprotein (LDL)–lowering atorvastatin therapy.^1^ By contrast, smaller randomized controlled trials of other CETP inhibitor agents (i.e., torcetrapib, dalcetrapib, and evacetrapib) that did not continue for as long were not able to demonstrate overall beneficial effects on major cardiovascular events.^2–4^ However, it has been suggested that the *ADCY9* genotype may identify individuals who would benefit particularly from CETP inhibitor therapy.^5,6^ In retrospective analyses of the dal-OUTCOMES trial, which involved 787 major cardiovascular events, there appeared to be a pharmacogenetic interaction, with a 39% proportional risk reduction in events in individuals assigned dalcetrapib who were homozygous for the rs1967309 A allele but with little apparent benefit among heterozygotes and a 27% increase in risk in those who were homozygous for the G allele. These observations, along with supporting evidence on carotid intima-media thickness, C-reactive protein, and cholesterol efflux suggesting a CETP-related mechanism linked to inflammation,^5,6^ prompted the initiation of the ongoing dal-GenE trial among individuals with evidence of recent acute coronary syndrome who are homozygous for the rs1967309 A allele.^7^ Following publication of a nonsignificant (albeit consistent) trend in clinical risk reductions with evacetrapib across rs1967309 genotypes (despite being driven by differences in the event rates between genotypes in the placebo arm) in a nested-case control pharmacogenetic study involving 1427 cardiovascular events in the ACCELERATE trial (Assessment of Clinical Effects of Cholesteryl Ester Transfer Protein Inhibition With Evacetrapib in Patients at a High Risk for Vascular Outcomes),^8^ the size of the dal-GenE study was increased from 5000 to 6000 randomized individuals, and it is currently anticipated to complete in late 2020.

The present pharmacogenetic study involving about 2500 major vascular events among more than 19 000 participants in the REVEAL trial includes a greater number of events than the 2 previous studies combined. Consequently, it provides a well-powered test of the hypothesis that *ADCY9* influences the effects of the CETP inhibitor anacetrapib on major cardiovascular outcomes.

## Methods

Proposals for data access will be considered by the REVEAL Steering Committee in accordance with the trial protocol. The procedures for accessing the data are available at https://www.ndph.ox.ac.uk/data-access.

### The REVEAL Trial

REVEAL was a randomized, double-blind, placebo-controlled trial of the CETP inhibitor anacetrapib, details of which have been reported previously.^1,9^ In summary, 30 449 men and women older than 50 years of age were recruited between 2011 and 2013 in Europe, North America, and China. Individuals were eligible if they had a history of myocardial infarction, cerebrovascular atherosclerotic disease, peripheral artery disease, or diabetes mellitus with symptomatic coronary heart disease. Individuals with an acute coronary event or stroke less than 3 months before randomization or with a planned coronary revascularization were excluded. After an 8 to 12-week prerandomization run-in phase with study atorvastatin alone, eligible patients were randomized to receive the addition of anacetrapib 100 mg once daily or matching placebo for a median duration of about 4 years. The prespecified primary outcome of the main trial was first major coronary event (a composite of coronary death, myocardial infarction, or coronary revascularization), with additional secondary outcomes including first major vascular event (a composite of major coronary event and presumed ischemic stroke). Adjudication was complete for 99.9% of primary and secondary outcomes. The trial was approved by all relevant institutional review boards and regulatory authorities, and participants provided written informed consent.

### Genotyping Assays

All randomized participants with appropriate consent from Europe and North America were included in the genetic substudy (permission to genotype participants recruited in China is not currently available). DNA was extracted from stored buffy coat or plasma-depleted blood at the UK Biocentre in the United Kingdom, and genotyping was undertaken by Thermo Fisher (Santa Clara, CA) using the Axiom Precision Medicine Research Array, which included direct measurement of the *ADCY9* rs1967309 variant. After exclusion of poor-quality DNA samples, genotyping was successful in 20 265 of the 20 395 participants in whom DNA was available. Subsequently, 19 951 individuals passed bioinformatic quality control and relatedness exclusions. To avoid the potential biases that can result from population stratification, the primary analyses were conducted among the 19 245 participants of European ancestry (based on concordant genetically determined and self-reported information). The genotype call rate for the *ADCY9* variant of interest (rs1967309) was 99.8%, which corresponds to directly measured genotype data for this variant being available in 19 210 participants.

### Statistical Analyses

Primary analyses in this REVEAL pharmacogenetic study are for major vascular events (which includes stroke) for greater comparability with previous studies that examined the potential *ADCY9* pharmacogenetic interaction.^6,8^ Intention-to-treat analyses were undertaken using Cox proportional hazards models to test for interactions between *ADCY9* genotype and the effects of anacetrapib on first major vascular event (and its components). Hazard ratios (HRs) were estimated from models with main effects for randomized group and for genotype (coded 0, 1, and 2, corresponding to the A allele count) and adjusted for the first 5 principal components of ancestry, with an interaction term between randomized group and genotype. Treatment-by-genotype interaction *P* values were obtained from 1 and 2 df likelihood ratio tests for additive (*P*_add_, per A allele) and genotypic (*P*_geno_, comparing AA versus AG versus GG) effects, respectively. The results from the previous hypothesis-testing trials were combined in an inverse-variance weighted fixed effects meta-analysis. Lipid and blood pressure differences between the randomized treatment groups were assessed at the trial midpoint, stratified by *ADCY9* genotype. Treatment-by-genotype interaction *P* values for the effects on lipids and blood pressure were obtained from 1 and 2 df likelihood ratio tests for additive and genotypic effects, respectively, based on modeling measurements at the trial midpoint adjusted for the first 5 principal components of ancestry and the comparable measurement at the randomization visit. All analyses were performed using SAS version 9.3 and R version 3.0.1.

## Results

### Baseline Characteristics

The frequency of the rs1967309 A allele was 39.8% overall (40.1% in the placebo group and 39.4% in the anacetrapib group), which is consistent with previous studies,^6,8^ and it did not deviate from Hardy–Weinberg equilibrium (p=0.93). Baseline characteristics in the genetic study did not differ materially from those in the whole trial (with the exception of race).^1^ Of the participants with *ADCY9* data, 86.0% were male, 86.6% had stable coronary heart disease (exclusion criteria included acute coronary event, stroke less than 3 months before randomization, or planned coronary revascularization), and 32.7% were diabetic. At randomization, LDL cholesterol levels were well controlled (1.6 mmol/L), with 51.0% of participants receiving atorvastatin 80 mg daily, while the remainder received 20 mg daily. These participant characteristics did not differ materially by *ADCY9* genotype (Table 1).

**Table 1.**
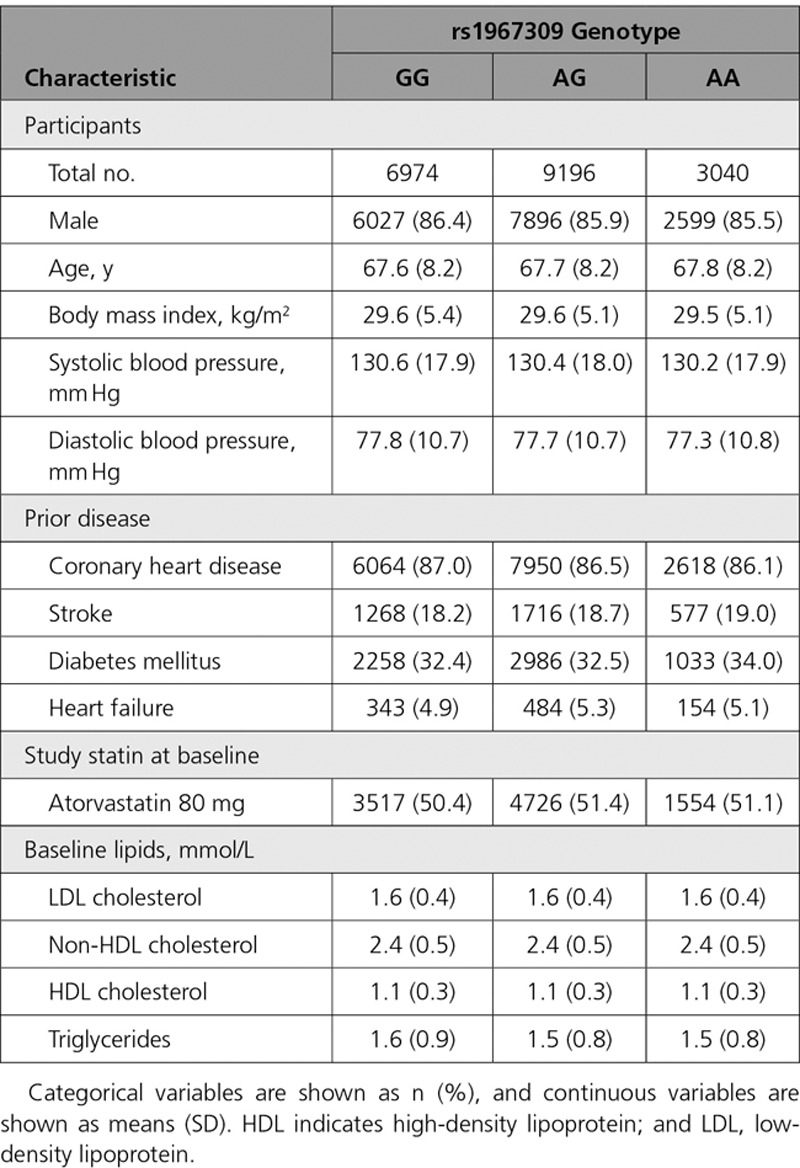
Baseline Characteristics of Randomized Participants, by ADCY9 Genotype

### Association of *ADCY9* Genotype With Major Vascular Events in Placebo-Allocated Participants

The association of *ADCY9* with incident major vascular events (ie, after randomization) was assessed in 9577 placebo-allocated participants. No significant association with *ADCY9* genotype was observed with either major vascular events (N=1288; *P*_add_=0.85, *P*_geno_=0.90; Table in the online-only Data Supplement) or major coronary events (N=1093; *P*_add_=0.93, *P*_geno_=0.69), irrespective of the modeling approach.

### Effects of Anacetrapib on High-Density Lipoprotein and Non–High-Density Lipoprotein Cholesterol, by *ADCY9* Genotype

Differences in non–high-density lipoprotein (HDL) cholesterol and HDL cholesterol between randomized groups were examined in strata defined by *ADCY9* genotype. The overall reduction in non-HDL cholesterol at the study midpoint was 0.45 mmol/L (18.1%), with a slightly larger absolute reduction in AA carriers than in others (Table 2). The interaction between anacetrapib and *ADCY9* genotype on non-HDL cholesterol was marginally significant after adjustment for principal components of ancestry alone (interaction *P*_add_=0.03) but was attenuated after further adjustment for non-HDL cholesterol levels at randomization (interaction *P*_add_=0.30, interaction *P*_geno_=0.34). Overall, there was a 1.12 mmol/L (100.4%) increase in HDL cholesterol with allocation to anacetrapib at the study midpoint, with no evidence of a treatment by *ADCY9* genotype interaction before or after adjustment for HDL cholesterol at randomization (Table 3).

**Table 2.**
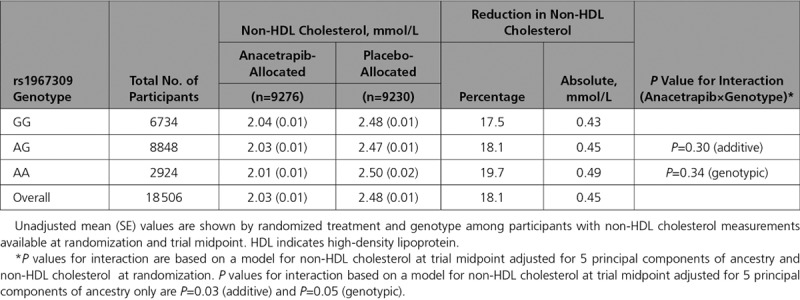
Effects of Anacetrapib on Non-HDL Cholesterol at Trial Midpoint, by ADCY9 Genotype

**Table 3.**
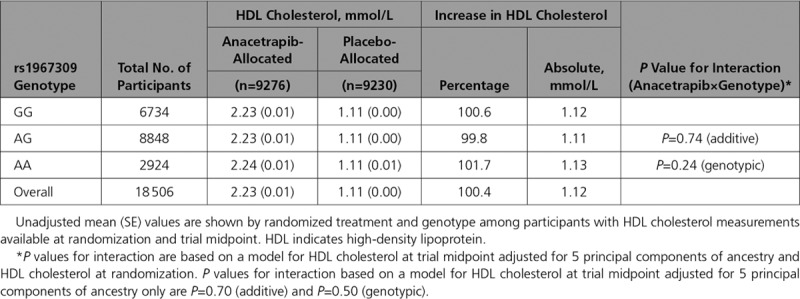
Effects of Anacetrapib on HDL Cholesterol at Trial Midpoint, by ADCY9 Genotype

### Effects of Anacetrapib on Systolic and Diastolic Blood Pressure, by *ADCY9* Genotype

Given previously observed associations between CETP inhibitor therapy and blood pressure, differences in systolic and diastolic blood pressure between randomized groups at the trial midpoint were examined in strata defined by *ADCY9* genotype (Table in the online-only Data Supplement). There was a 0.75 mm Hg higher systolic blood pressure among participants allocated anacetrapib versus placebo overall but no significant interaction with *ADCY9* genotype for either systolic or diastolic blood pressure.

### Effects of Anacetrapib on Major Vascular Events, by *ADCY9* Genotype

**Figure 1.**
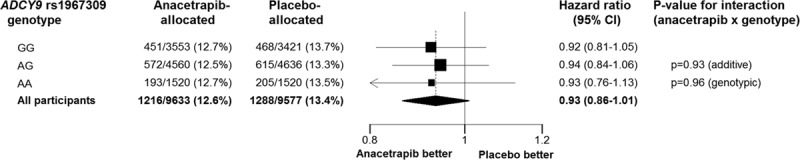
**Effects of anacetrapib on major vascular events, by *ADCY9* genotype.** Hazard ratios for each genotype are indicated by squares (size inversely proportional to the variance) with horizontal lines indicating 95% CIs. The hazard ratio and 95% CIs for all participants combined is indicated by a diamond.

Among the genotyped individuals, 2504 (13.0%) had a major vascular event during the 4-year median follow-up period: 1216 (12.6%) among anacetrapib-allocated participants and 1288 (13.4%) among placebo-allocated participants. There was a 7% proportional reduction in major vascular events (HR = 0.93; 95% CI, 0.86–1.01), consistent with the effect of anacetrapib on major vascular events observed in the whole trial (HR = 0.93; 95% CI, 0.88–0.99). As illustrated in Figure 1, there was no evidence that the proportional reduction in major vascular events was associated with rs1967309 genotype status: HR = 0.92 (95% CI, 0.81–1.05) for GG; HR = 0.94 (95% CI, 0.84–1.06) for AG; and HR = 0.93 (95% CI, 0.76–1.13) for AA; interaction *P*_add_=0.93; interaction *P*_geno_=0.96.

Analyses stratified by baseline characteristics among participants with or without coronary heart disease, with or without diabetes mellitus, receiving atorvastatin 80 mg or 20 mg daily, and male or female did not identify an impact of *ADCY9* genotype on the proportional reduction in major vascular events (all allelic and genotypic interaction *P* values of >0.05). Nor were there significant differences between the proportional reductions in any of the major vascular event components (Figure I in the online-only Data Supplement). Further more, in a sensitivity analysis including all 19 912 participants with *ADCY9* genotype data passing quality control (ie, not restricted to those of European ancestry), there was also no impact of *ADCY9* genotype on the proportional risk reduction in major vascular events (interaction *P*_add_=0.96, interaction *P*_geno_=0.87).

## Discussion

These pharmacogenetic data from the REVEAL trial provide results from the single largest study to date of the impact of *ADCY9* on the effects of CETP inhibitor therapy. They do not support the hypothesis that the effects on major vascular events of CETP inhibitor therapy (at least with anacetrapib) differ among people with different *ADCY9* genotypes. The large number of events on which these analyses are based enable us to exclude the hypothesized large differences in the proportional risk reductions between *ADCY9* genotype groups.

### *ADCY9* and CETP Inhibition

Adenylyl cyclase is a membrane-bound enzyme that catalyses the formation of cyclic AMP from ATP. The *ADCY9* gene encodes adenylate cyclase type 9, which is a widely distributed adenylyl cyclase, and the rs1967309 variant has been associated with changes in *ADCY9* gene expression.^10^ Potential mechanisms linking *ADCY9* and CETP inhibition remain unclear. However, further to the observation of an apparent difference in the effect of dalcetrapib on cardiovascular events in people with different *ADCY9* genotypes, some supporting evidence has emerged. For example, among 386 participants in the dal-PLAQUE-2 trial, assignment to dalcetrapib for 6–12 months was associated with plaque regression among individuals with the AA genotype but not among those with other rs1967309 genotypes.^6^ It has also been suggested that *ADCY9* may modulate immune cell function and inflammatory responses.^11,12^ In dal-OUTCOMES, there was an overall 18% proportional increase in C-reactive protein between baseline and the trial end but no change in those with the AA genotype (*P*=0.02 for interaction).^5^ In contrast, however, the 9% proportional increase in C-reactive protein observed in the ACCELERATE trial was not influenced by *ADCY9* genotype (data on C-reactive protein are not currently available for REVEAL). Experimental studies in mice have indicated that, in the absence of CETP activity, *ADCY9* inactivation protects against atherosclerosis, potentially through decreased macrophage accumulation and proliferation in the arterial wall, as well as by improving endothelial function.^13^ *ADCY9* mediates β2-adrenoceptor signaling, and it has been suggested that an interaction between HDL/ApoA1 and *ADCY9* on this signaling may be relevant to the apparent pharmacogenetic interaction observed in dal-OUTCOMES.^14^

### Comparison With Previous Trials of CETP Inhibition

REVEAL involved a considerably longer treatment period than did dal-OUTCOMES and ACCELERATE (over 4 years vs 2–2.5 years) and is the only study of a CETP inhibitor to have demonstrated beneficial effects on clinical outcomes (Table 4). The lipid effects of the various CETP inhibitors differ materially: both evacetrapib and anacetrapib increase HDL cholesterol by more than 100% as well as reducing non-HDL cholesterol by ~20%; by contrast, dalcetrapib increases HDL cholesterol by ~30% and has no effect on non-HDL cholesterol. In addition, the underlying study populations differed somewhat, with dal-OUTCOMES enrolling patients 4–12 weeks after an acute coronary syndrome, ACCELERATE enrolling patients 1–12 months after an acute coronary syndrome, and REVEAL excluding patients within 3 months of an acute coronary syndrome (as well as enrolling patients with cerebrovascular disease, peripheral arterial disease, or diabetes mellitus with symptomatic coronary heart disease). The number of events included in the REVEAL pharmacogenetic study was over 3-fold greater than that in dal-OUTCOMES and 75% greater than that in ACCELERATE.

**Table 4.**
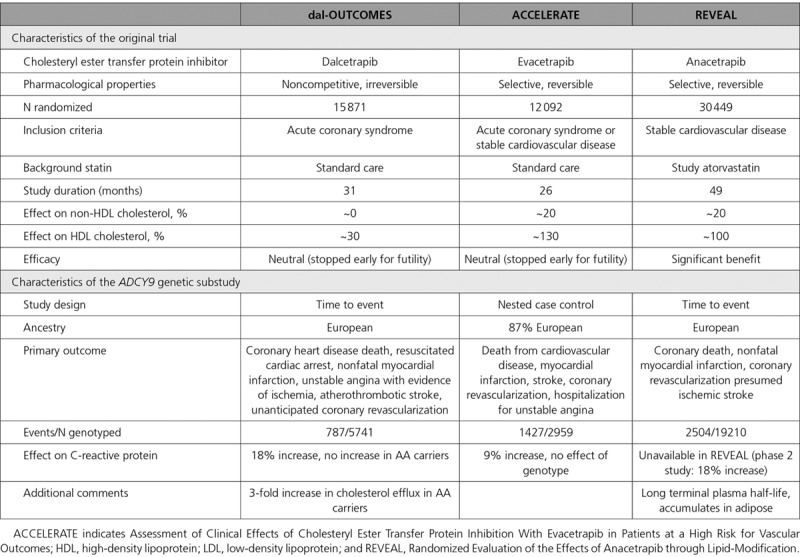
Comparison of Characteristics Between Cholesteryl Ester Transfer Protein Inhibitor Trials Examining Clinical Response, by ADCY9 Genotype

CETP inhibition is known to lower non-HDL cholesterol levels by decreased transfer of HDL cholesteryl ester into triglyceride-rich lipoproteins that are converted into LDL, decreased transfer of HDL cholesteryl ester into LDL, and increased uptake of LDL particles by the hepatic LDL receptor.^15^ Both evacetrapib and anacetrapib reduce LDL cholesterol, with the effect of anacetrapib on clinical events in REVEAL consistent with that expected from the achieved change in non-HDL cholesterol over a 4-year period. In REVEAL, there was no material difference in the effects of anacetrapib on these biochemical measures by *ADCY9* genotype. By contrast, although dalcetrapib has little overall effect on LDL cholesterol, there was an apparent trend towards a slightly larger reduction in LDL cholesterol among those with the rs1967309 AA genotype in dal-OUTCOMES.^6^

**Figure 2.**
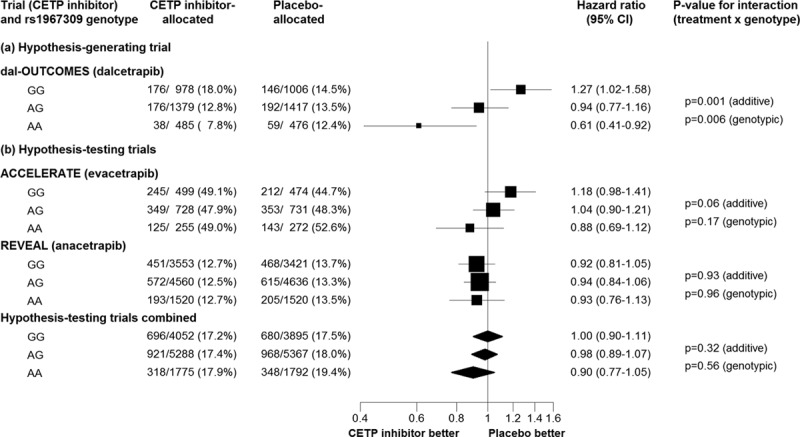
**Clinical response to CETP inhibition in hypothesis-generating and hypothesis-testing trials, by *ADCY9* genotype.***P* values are taken from published materials for individual studies and from chi-square tests for trend and heterogeneity across genotypes for the combined hypothesis testing studies. dal-OUTCOMES events include coronary heart disease death, resuscitated cardiac arrest, nonfatal myocardial infarction, atherothrombotic stroke, unstable angina with evidence of ischemia or unanticipated coronary revascularization. ACCELERATE events include cardiovascular death, myocardial infarction, stroke, coronary revascularization, or hospitalization for unstable angina. REVEAL events include coronary death, myocardial infarction, coronary revascularization or presumed ischemic stroke. ACCELERATE indicates Assessment of Clinical Effects of Cholesteryl Ester Transfer Protein Inhibition With Evacetrapib in Patients at a High Risk for Vascular Outcomes; CETP, cholesteryl ester transfer protein; and REVEAL, Randomized Evaluation of the Effects of Anacetrapib through Lipid-Modification.

Figure 2 shows the observed effects of CETP inhibitor therapy on major vascular events by *ADCY9* genotype in the original hypothesis-generating dal-OUTCOMES trial in which there was a 39% (95% CI, 8%–59%) proportional reduction with dalcetrapib in individuals with rs1967309 AA genotypes compared with a 27% (95% CI, 2%–58%) proportional increase in those with GG genotypes, yielding a significant interaction (interaction *P*_add_=0.001, interaction *P*_geno_=0.006). In the ACCELERATE genetic study, which involves high proportions of genotyped individuals with cardiovascular events because of its case-control design, there was a nonsignificant trend that is directionally consistent with the dal-OUTCOMES result. That trend appears to be driven largely by differences in the event rates in the placebo arm rather than by differential rates in the evacetrapib arm. Moreover, when the hypothesis-testing ACCELERATE and REVEAL trials are combined, *ADCY9* genotype is not associated with significant differences in the proportional reductions in major vascular events (interaction *P*_add_=0.32, interaction *P*_geno_=0.56). Given the *ADCY9*-dependent effects of dalcetrapib on C-reactive protein and efflux capacity that have not currently been observed with the other CETP inhibitors, it is still possible that the relevance of *ADCY9* genotypes to vascular risk reduction may be specific to dalcetrapib.

### Conclusions

Results from the REVEAL trial do not provide support for the hypothesis that *ADCY9* genotype is materially relevant to the effects of anacetrapib on major vascular events. The dal-GenE trial, which completed recruitment in late 2018 and has an estimated completion date of late 2020, will provide a specific test of the effects of dalcetrapib among people with the rs1967309 AA genotype.^7^

## Acknowledgments

The authors thank the REVEAL trial participants, the local clinical center staff, regional and national coordinators, and members of the steering committee, lipid monitoring committee, and data monitoring committee. A complete list of members of the HPS3/TIMI55–REVEAL Collaborative Group is provided in the online-only Data Supplement.

## Sources of Funding

This work was supported by grants from Merck & Co., Inc., to the University of Oxford. Support was also provided by the British Heart Foundation (including direct support for Dr Hopewell through grant FS/14/55/30806 and Dr Collins), the Medical Research Council (which funds the Medical Research Council Population Health Research Unit in a strategic partnership with the University of Oxford), the National Institute for Health Research Clinical Research Network, Health Data Research UK, and the National Institute for Health Research Oxford Biomedical Research Centre.

## Disclosures

Drs Hopewell, Ibrahim, Hill, Bowman, Landray, and Collins work at the Clinical Trial Service Unit & Epidemiological Studies Unit (CTSU), Nuffield Department of Population Health, University of Oxford. The Clinical Trial Service Unit & Epidemiological Studies Unit have a staff policy of not taking any personal payments directly or indirectly from industry (with reimbursement sought only for the costs of travel and accommodation to attend scientific meetings). The Clinical Trial Service Unit & Epidemiological Studies Unit has received research grants from Abbott, AstraZeneca, Bayer, Boehringer Ingelheim, GlaxoSmithKline, The Medicines Company, Merck, Mylan, Novartis, Pfizer, Roche, Schering, and Solvay, which are governed by University of Oxford contracts that protect their independence. Drs Shaw and Blaustein are employees of Merck & Co., Inc. Dr Braunwald reports grants to his institution from Merck, Daiichi Sankyo, AstraZeneca, GlaxoSmithKline, and Novartis; personal fees for consultancies with Theravance, Cardurion, Verve, and MyoKardia; personal fees for lectures from Medscape; and uncompensated lectures for The Medicines Company, Novartis, and Merck. Dr Sabatine reports research grant support through Brigham and Women’s Hospital from Abbott Laboratories; Amgen; AstraZeneca; Bayer; Daiichi-Sankyo; Eisai; Gilead; GlaxoSmithKline; Intarcia; Janssen Research and Development; Medicines Company; MedImmune; Merck; Novartis; Poxel; Pfizer; Quark Pharmaceuticals; Roche Diagnostics; Takeda, and consulting fees from Alnylam; Amgen; AstraZeneca; Bristol-Myers Squibb; CVS Caremark; Dyrnamix; Esperion; IFM Therapeutics; Intarcia; Ionis; Janssen Research and Development; Medicines Company; MedImmune; Merck; MyoKardia; and Novartis. Dr Collins is coinventor of a genetic test for statin-related myopathy risk but receives no income from it.

## Supplementary Material

## Appendix

### Members of the REVEAL Steering Committee

MJ Landray, Clinical Trial Service Unit and Epidemiological Studies Unit, Nuffield Department of Population Health, University of Oxford, UK. L Bowman, Clinical Trial Service Unit and Epidemiological Studies Unit and Medical Research Council Population Health Research Unit, Nuffield Department of Population Health, University of Oxford, UK. R Collins, Clinical Trial Service Unit and Epidemiological Studies Unit, Nuffield Department of Population Health, University of Oxford, UK. E Braunwald, TIMI Study Group, Cardiovascular Division, Brigham and Women’s Hospital, Boston, MA. JC Hopewell, Clinical Trial Service Unit and Epidemiological Studies Unit, Nuffield Department of Population Health, University of Oxford, UK. L Jiang, Department of Cardiology, Fuwai Hospital, National Center for Cardiovascular Diseases, Chinese Academy of Medical Sciences and Peking Union Medical College, Beijing, People’s Republic of China. CP Cannon, TIMI Study Group, Cardiovascular Division, Brigham and Women’s Hospital, Boston, MA. SD Wiviott, TIMI Study Group, Cardiovascular Division, Brigham and Women’s Hospital, Boston, MA. J Armitage, Clinical Trial Service Unit and Epidemiological Studies Unit and Medical Research Council Population Health Research Unit, Nuffield Department of Population Health, University of Oxford, UK. R Haynes, Clinical Trial Service Unit and Epidemiological Studies Unit and Medical Research Council Population Health Research Unit, Nuffield Department of Population Health, University of Oxford, UK. AP Maggioni, National Association of Hospital Cardiologists Research Center, Florence, Italy. CE Angermann, Comprehensive Heart Failure Center, University Hospital and University of Würzburg, Germany. G Ertl, Comprehensive Heart Failure Center, University Hospital and University of Würzburg, Germany. C Wanner, Division of Nephrology, University Hospital Würzburg, Würzburg, Germany.T Pedersen, Department of Medicine, Oslo University Hospital, Ullevål, Norway. S Goto, Department of Medicine (Cardiology), Tokai University School of Medicine, Japan. T Teramoto, Teikyo Academic Research Center, Teikyo University, Japan. C Baigent, Clinical Trial Service Unit and Epidemiological Studies Unit and Medical Research Council Population Health Research Unit, Nuffield Department of Population Health, University of Oxford, UK. P Barter, School of Medical Sciences, University of New South Wales, Sydney, Australia. Y Chen, Clinical Trial Service Unit and Epidemiological Studies Unit, Nuffield Department of Population Health, University of Oxford, UK. Z Chen, Clinical Trial Service Unit and Epidemiological Studies Unit, Nuffield Department of Population Health, University of Oxford, UK. A Gray, Health Economics Research Centre, Nuffield Department of Population Health, University of Oxford, UK. B Mihaylova, Health Economics Research Centre, Nuffield Department of Population Health, University of Oxford, UK; Centre for Primary Care and Public Health, Blizard Institute, Barts and the London School of Medicine and Dentistry, Queen Mary University of London, UK. P Sleight, Nuffield Department of Medicine, John Radcliffe Hospital, Oxford, UK. J Tobert, Nuffield Department of Population Health, University of Oxford, UK. R Blaustein, Merck Research Laboratories, Merck & Co., Inc., Kenilworth, NJ. P DeLucca, Merck Research Laboratories, Merck & Co., Inc., Kenilworth, NJ. Y Mitchel, Merck Research Laboratories, Merck & Co., Inc., Kenilworth, NJ. G van Leijenhorst, Merck Research Laboratories, Merck & Co., Inc., Kenilworth, NJ.
