# Supplementary material for: Impact of ADCY9 Genotype on Response to Anacetrapib
Source: Circulation. 2019 Jul 23;140(11):891–8. doi: 10.1161/CIRCULATIONAHA.119.041546 (PMC6749971; doi:10.1161/CIRCULATIONAHA.119.041546)
Supplement: Supplementary file 1 [file cir-140-891-s001.pdf]

SUPPLEMENTAL MATERIAL

Contents

Details of the REVEAL Collaborative Group **2**

Supplemental Tables and Figures **16**

Supplemental Table 1: Association of *ADCY9* genotype with components of major vascular events among placebo-allocated participants

Supplemental Table 2: Effects of anacetrapib on blood pressure at trial midpoint, by *ADCY9* genotype

Supplemental Figure 1: Effects of anacetrapib on components of major vascular events, by *ADCY9* genotype

Details of the REVEAL Collaborative Group

Steering Committee

MJ Landray, L Bowman (Principal Investigators); R Collins (Chair); E Braunwald (Deputy Chair); JC Hopewell (Trial Statistician); L Jiang, CP Cannon, SD Wiviott, J Armitage, R Haynes, AP Maggioni, CE Angermann, G Ertl, C Wanner, T Pedersen, S Goto, T Teramoto (Regional Representatives); C Baigent, P Barter, Y Chen, Z Chen, A Gray, B Mihaylova, P Sleight, J Tobert (other voting members); R Blaustein, P DeLucca, Y Mitchel, G van Leijenhorst (non-voting Merck representatives)

Data Monitoring Committee

P Sandercock (Chair), D DeMets, J Kjekshus, J Neuberger, A Tonkin; J Emberson[+] ([+]non-voting DMC Statistician)

Lipid Monitoring Committee

C Granger (Chair), H Colhoun; K Wallendszus (non-voting statistical programmer)

Coordinating Centres

Central Coordinating Office and UK (Clinical Trial Service Unit, University of Oxford):
Management Committee: MJ Landray, L Bowman (principal investigators), J Barton, C Bray, R Dayanandan, C Knott, M Lay, K Murphy, E Wincott; *Administration and support:* J Barton, C Bray, R Dayanandan, K Murphy, E Wincott (coordinators), P Achiri, S Barry, S Bateman, A Brewster, S Briggs, R Brown, A Burke, E Butler, L Cobb, A Collet, J Crowther, L Cureton, S Danesh-Pour, S Fathers, L Fletcher, K Frederick, T Gordon, M Gray, J Heineman, S Howard, D Jackson, N Lam, R Lee, O Machin, Z Madgwick, M Matthewson, J Nolan, M Nunn, A Panicker, L Pank, E Pearson-Burton, S Pickworth, Y Qiao, A Radley, K Roby, J Sayer, S Shah, K Taylor, H Thorne, A Timadjer, K Vandenberg, M Wickman, M Willett, J Woods, H Yu; *Clinical support and adjudication:* J Armitage, T Aung, L Bowman, R Bulbulia, F Chen, R Clarke, R Haynes, W Herrington, P Judge, MJ Landray, D Lewis, R Llewellyn-Bennett, M Mafham, D Preiss, C Reith, E Sammons, B Storey, J Tomson, E Waters; *Computing & validation:* A Baxter, M Lay, R Goodenough (coordinators); R Ait-Sadi, M Arnold, I Barton, C Berry, G Blower, J Booth, E Brown, Y Bu, P Cleverley, G Coates, J Cox, M Craig, G Cui, P Dalton, L Danel, C Daniels, C Dawe, A Field, S Gilbert, P Harding, K Jayne, R Kurien, G Lancaster, A Maskill, A McDougall, Y Mostefai, S Mulay, A Munday, A Murawska, N Prajapati, S Ramesh, R Reid, S Syed, H Todd, A Young, A Young, W Zhu; *Statistical analysis:* JC Hopewell (study statistician), K Wallendszus (statistical programming coordinator), M Arnold, S Parish, W Stevens, E Valdes-Marquez; *NDPH Wolfson Laboratory:* M Hill (laboratory director), S Clark, K Emmens, G McClean, M Radley, J Wintour (laboratory coordinators); M Allworth, A-M Beneat, C Bird, L Boggs, A Casey, T Chavagnon, K Chung, R Chung, L Cockram, R Cox, J Douglas, L Finnegan, H French, N Goodwin, A Gordon, J Gordon, C Guest, S Hazim, J Hill, R Hrusecka, M Lacey, N Luker, S Mulligan, ME Obrero, N Plunkett, L Sansom, R Shellard, J Taylor, P Taylor, J Tyler, L Weaving, J Wheeler, T Williams, M Yeung; *Monitors and trainers:* C Knott (coordinator); S Beebe, K Bowsher-Brown, J Dabrowski, J Henderson, J James, H Lochhead, V Toghill, L Wright, L Young;

China (China-Oxford Centre for International Health Research, Fuwai Hospital, Beijing):
Regional coordinator: L Jiang; *Clinical support and adjudication:* W Hundei, J Liu, J Qu, H Zhang; *Administration and support:* H Dai, F Feng, L Hou, J Li, L Ma, S Niu, R Tang, S Wang, X Wei, M Xie, X Yan, M Yang, Y Zhang, L Zhang, A Zhang, S Zhang, L Zhao, H Zhong; *Monitors and Trainers:* L Chen, Y Gao, L Li, H Yang; *IT Support:* J Zhang;

Germany (Comprehensive Heart Failure Center [CHFC] and Department of Internal Medicine I, University Hospital, Würzburg):
Regional coordinators: CE Angermann, G Ertl, C Wanner; *Clinical support and adjudication:* S Brenner, M Heldmann, B Kraus, B Meyer; *Administration and support:* M Fajardo-Moser, C Hartner; *Monitors and Trainers:* A Knoppe, D Pop-Marschall, J Renner, U Saemann;

Italy (Centro Studi Associazione Nazionale Medici Cardiologi Ospedalieri [ANMCO], Florence):
Regional coordinator: AP Maggioni; *Clinical support and adjudication:* G Fabbri; *Administration and support:* A Lorimer, D Lucci, B Bartolomei Mecatti; *Monitors and Trainers:* M Ceseri, E Baldini, S Benoni, F Bianchini, P Ferruzzi, M Miccoli, S Musio, F Ramani; *IT Support:* M Gorini, G Orsini;

Japan (School of Medicine Multiregional Study Office, Tokai University):
Regional coordinators: S Goto, T Teramoto; *Clinical support and adjudication:* E Kato, K Tawara, A Tomita; *Administration and support:* S Kitamura, Y Saitoh, M Shimizu, S Shiozaki, K Soeda, A Tanaka; *Monitors and Trainers:* E Kato; K Tawara; A Tomita;
[Note: These collaborators participated in preparations to initiate recruitment in Japan, but it was subsequently planned not to extend recruitment there.]

North America (TIMI Study Group, Boston):
Regional coordinators: E Braunwald, CP Cannon, SD Wiviott; *Clinical support and adjudication:* A Eisen, E Kato, D Steen; *Administration and support:* P Fish (director of operations), S MacDonnell, J Kent, A McCagg (lead coordinators), E Greene, D Klements, K Washington; *Monitoring and training (Covance, Princeton):* A Davis, M Goeres, J Joyce, J Koen (lead monitors), J Colicchia, J Domercant, V Foster, C Fox, C Gennusa, R Hollis, Y Kassa, A Kelley, V Magloire, C Owens, N Yeh;

Scandinavia (MSD, Copenhagen):
Regional coordinator: T Pedersen (*Ullevål Universitetssykehus HF, Oslo*); *Coordinators:* K Arnesson, S Mosegaard; *Clinical support and adjudication:* K Andersen, S Haywood, A Osmanagic, C Pilgaard Madsen, E Rebnord, K Serup-Hansen, M Tarras Wahlberg; *Administration and support:* Denmark: K Hannibal, T Johansen, L Rasmussen, A Sloth; Finland: P Kiuru, M Lauronen, A-S Leinonen, T Mononen, M Vuola, S Wiik; Norway: H Hovdal, C Lien, S Svingen, P Singh, I Thorsby, E Westerheim; Sweden: P Bergsten, L Bergvall, H Castedal, A-C Cederholm, L Froberg, A Johansson, L Jonsson, P Martin, T Rasmusson, S Wiik-Karu; *Monitoring and training:* Denmark: H Diget, O Moll, S Snejbjerg, G Sørensen; Finland: S Eronen, S Roine, T Vaine; Norway: V Bjørhovde, L Edvardsen, S Saether; Sweden: Å Blechert, I Ek, L Hedlöf, J Levin, D Vlaheli;

Local Clinical Centres

Canada

National coordinator: J Genest; *Collaborators: Brampton Research Associates, Brampton, ON:* M Gupta, A Burgess, C Dela Cruz, S Harnden, S Hirjikaka, E Mallari, Y Thevakumaran; *Cambridge Cardiac Care Inc., Cambridge, ON:* A Pandey, J Lake, M Pandey, C Wang; *Centre de Dépistage et Recherche Cardiovasculaire Rive-Sud, Longueuil, QC:* E Sabbah, I Chausse, F Deslongchamp, J Lavoie; *Centre de Recherche Medialpha, St. Lachine, QC:* G Sabe-Affaki, S Fontaine; *Centre intégré de santé et de services sociaux de Lanaudière - Hôpital Pierre-Le Gardeur, Terrebonne, QC:* G Gosselin, M David, K Drouin, N Lachance, C Masson, M Pashko, C Tremblay; *Clinique Sante Cardio MC, Montreal, QC:* C Constance, M Gauthier; *CSSSNL/CHRDL, Saint-Charles-Borromee, QC:* S Kouz, C Fleury, V Lemay-Chretien, N Roberge, M Roy; *Discovery Clinical Services LTD, Victoria, BC:* G Hoag, R Standring, L Warke; *Dr. Stephen Pearce, Inc, Surrey, BC:* S Pearce, L Breakwell, T Cleveland, D Kastanis; *Heart Care Research, Oshawa, ON:* R Bhargava, C Stafford, C Stata; *Heritage Medical Research Clinic, Calgary, AB:* T Anderson, D Brown, B Madden, M Pajevic, D Ramadan, B Smith; *James Cha MD, Oshawa, ON:* J Cha, J Otis; *Newmarket Cardiology Research Group, Newmarket, ON:* R Zadra, A Harwood, C McPherson, C Rackham; *Oshawa Clinic, Oshawa, ON:* A Bakbak, S Baghiana, K Gibney, L Swailes; *Q&T Research Outaouais Incorporated, Gatineau, QC:* P Nault, K Audet, C Roy, E St-Amour, I Tremblay; *Viacar Recherche Clinique Inc., Greenfield Park, QC:* R Chehayeb, C Lepage; Vizel Cardiac Research, Cambridge, ON: S Vizel, B Fox;

China

National coordinator: L Jiang; *Collaborators: Affiliated Zhongshan Hospital of Dalian University, Department of Cardiology:* Q Yu, L Chi, F Liu; *Baotou Central Hospital, Department of Cardiology:* R Zhao, X Li, Y Qian, J Wang; *Beijing Chao-Yang Hospital, Capital Medical University, Department of Cardiology:* X Yang, M Chen, X Lin, F Zhang; *Beijing Liangxiang Hospital of Fangshan District, Department of Cardiology:* X Fang, Q Yu, W Su, X Zhu; *Changsha Central Hospital, Department of Cardiology:* H Dai, L Huang, G Ye, Y Zhao; *China-Japan Union Hospital of Jilin University, Department of Cardiology:* P Yang, M He, B Li; *Dalian Municipal Central Hospital, Division of Cardiology:* Y Zhi, L Sun, L Xiao, Y Yuan; *Fenyang Hospital of Shanxi Province, Department of Cardiology:* R Guo, Q Wang, Y Wang; *Fuwai Hospital, 25th Ward:* J Li, Y Gao, Y Guo, L Li, M Zhai, L Zhang; *Fuwai Hospital, Department of Cardiology:* Y Yang, J Song; *Fuwai Hospital, Department of Heart Surgery:* Z Zheng, X Wang, Y Zhao, C Zhao; *Houma City People Hospital, Department of Cardiology:* Z Wang, C Li; *Hulun Buir People's Hospital, Department of Geriatrics:* Z Cui, X Zhang, L Zhao*; Inner Mongolia Autonomous Region of Traditional Chinese-Mongolian Medicine Hospital, ICU:* H Su, X Huang, R Zhang; *Inner Mongolia Baogang Hospital, Department of Cardiology:* Z Ge, D Liu, Q Liu; *Inner Mongolia People's Hospital, Department of Cardiology:* Y Han, W He, Y Zhang; *Jilin City Central Hospital, Department of Cardiology:* D Qian, L Liu, X Yao; *Jilin Province People's Hospital, Department of Endocrinology:* Y Du, L Song; *Liaoning Shenyang Sujiatun District Central Hospital, Department of Cardiology:* H Che, D Li, C Sun; *Peking Union Medical College Hospital, Department of Cardiology:* S Zhang, H Bai, W Chen, Y Han, Z Liu, J Yang; *Peking University Third Hospital, Department of Cardiology:* Z Li, J Bai, F Wang; *Qingdao Municipal Hospital, Department of Cardiology:* X Wang, C Xing, Y Yao; *Qingdao Fuwai Hospital, Chinese Academy of Medical Science, Department of Cardiology:* X Jiang, Y Yang, Y Dong, G Wu, B Zhang; *Shanghai*

Changzheng Hospital, Department of Cardiology: Z Wu, W Chen, Y Chu, X Gu; *Shanxi Cardiovascular Hospital, Department of Cardiology:* B Li, J Wang, X Chai, H Zhang; *Shanxi Provincial People's Hospital, Department of Cardiology:* H Zhang, S Sun, L Tong; *Shengjing Hospital of China Medical University, Department of Cardiology:* X Li, S Ma, H Li, J Liu, X Liu, J Shi; *Shuangshan Hospital of Anshan, Department of Cardiology:* R Xiao, X Li, R Wu; *Suzhou Kowloon Hospital, Shanghai Jiaotong University Medical School, Department of Cardiology:* F Liu, X Meng, B Shao, T Zhang; *Tai Yuan City Centre Hospital, Department of Cardiology:* X Chen, T Feng, L Huo, X Shang; *The Affiliated Hospital of Medical College Qingdao University, Department of Emergency Cardiology:* C Zhou, M Guo, P Li; *The Affiliated Hospital of Medical College Qingdao University, Department of Emergency Neurology:* H Pei, D Han, H Li; *The Affiliated Hospital of Xuzhou Medical College, Department of Cardiology:* D Li, C Cheng, M Huang, W Wu, T Xu; *The Affiliated People's Hospital of Inner Mongolia Medical College, Cadre Ward:* J Liu, J Xia; *The Central Hospital of Wuhan, Department of Endocrinology:* S Zhao, C Cheng, S Ding, L Guo, N Li; *The Fifth People's Hospital of Shenyang, Department of Cardiology:* Q Diao, Z Liu, H Wang; *The First Affiliated Hospital of China Medical University, Department of Cardiology:* G Qi, Z Jia, Y Meng, C Wu; *The First Affiliated Hospital of China Medical University, Department of Cardiology, Anshan Hospital:* B Liu, X Bian; *The First Affiliated Hospital of Harbin Medical University, Department of Cardiology:* W Li, M He, J Jing, B Liu; *The First Affiliated Hospital of Harbin Medical University, Department of Neurology:* L Zhang, Y Sun, X Wang, S Wu, Y Xu; *The First Affiliated Hospital of Xinxiang Medical University, Department of Cardiology:* F Lv, C Guo, J Long, Y Wang; *The First Affiliated Hospital of Zhengzhou University, Department of Cardiology:* Z Huang, X Fu, H Yao, L Zhang; *The First Hospital of Jilin University, Department of Cardiology:* Y Zheng, X Li, C Liu, Q Tong; *The First Hospital of Jilin University, Department of Endocrinology:* Q Liu, G Wang, Y Cheng, X Gang, W Guo, G He; *The First Hospital of Shanxi Medical University, Department of Cardiology:* Q Han, H Bian, L Duan, C Jin, X Wei; *The First People's Hospital of Shenyang, Department of Cardiology:* F Feng, W Xing, J Xu; *The Fourth Affiliated Hospital of China Medical University, Department of Cardiology:* Y Jin, Y Lin, X Zhou; *The Fourth People's Hospital of Shenyang, Department of Cardiology:* Y Li, X Guan, X Zhou; *The General Hospital Of AISCO, Department of Cardiology:* X Liu, H Liu, L Liu; *The General Hospital of FAW, Department of Cardiology:* H Pan, X Wang, S Zhang; *The General Hospital of Shenyang Military Command, Department of Cardiology:* Y Han, P Fan, J Li, R Ma, G Wang, P Wang; *The General Hospital of Xuzhou Mining Group, Department of Cardiology:* W Wu, L Li; *The People's Hospital of Liaoning Province, Department of Cardiology:* Z Li, R Cui; *The Second Affiliated Hospital of Baotou Medical College, Department of Cardiology:* G Sun, F Wang, R Xie; *The Second Affiliated Hospital of Dalian Medical University, Cardiovascular Department:* P Qu, G Li, J Mei, L Wang, Q Yang, C Zhang; *The Second Affiliated Hospital of Harbin Medical University, Department of Cardiology:* B Yu, W Cao, W Du, Y Luan; *The Second Affiliated Hospital of Harbin Medical University, Department of Neurology:* W Wang, Y Zhu, H Jiao, Y Qu, Y Zhu; *The Second Hospital of Shanxi Medical University, Department of Cardiology:* Z Yang, N Du, J Li, B Liang, W Wu, H Yang; *The Second Hospital of Tianjin Medical University, Department of Neurology:* X Li, D Wang, P Zhao; *The Third People's Hospital of Dalian, Department of Cardiology:* N Li, X Liu, B Xu, D Zong; *The Third People’s Hospital of Xuzhou, Department of Cardiology:* L Wang, X Tang, C Zong; *The Third Xiangya Hospital of Central South University, Department of Endocrinology:* Z Mo, P Jin, J Xiong; *Tianjin Chest Hospital, Department of Cardiology:* H Cong, X Guo, R Liang, J Zhou; *Tianjin*

Fourth Center Hospital, Department of Cardiology: H Zhang, Y Liu, Z Sun; *Tianjin Medical University General Hospital, Department of Cardiology:* Y Sun, Z Wan, B Bian, Y Li, W Zhang; *Tianjin Union Medicine Centre, Department of Cardiology:* Z Yao, Y Liu, R Wang; *Tianjin Union Medicine Centre, Department of Neurology:* C Zhang, G Chen, C Ma; *Tongji Hospital, Department of Cardiology, Tongji Medical College:* D Wang, J Jiang, L Ni, H Yan; *Wuhan Asia Heart Hospital, Department of Cardiology:* X Su, J Cai, J Ma, R Zhong; *Wuhan Puai Hospital, Department of Cardiology:* Y Gu, L Hu, X Wu; *Wuxi People's Hospital, Department of Cardiology:* X Wu, Z Yang, M Chen, J Feng; *Xiangtan City Central Hospital, Department of Cardiology:* H Huang, F Ouyang, Z Sun, Y Zhou; *Xiangya Hospital Central-South University, Department of Cardiology:* T Yang, X Deng, L Peng, Y Zhao; *Xinxiang Central Hospital, Department of Cardiology:* L Liu, S Su; *Xuzhou Central Hospital, Department of Cardiology:* Q Fu, L Wang, X Zong; *Xuzhou N° 1 People's Hospital, Department of Cardiology:* H Zhang, L Li, X Liu, Y Shang; *Zhengzhou Central Hospital, Cardiovascular Medicine Department:* L Zhang, H Li, H Sun; *Zhu Zhou N° 1 Hospital, Department of Cardiology:* S Guo, Y He, L Cha, Y Lu;

Denmark
National coordinator: K Egstrup; *Collaborators: Aalborg Hospital:* E Berg Schmidt, P Dinesen, A Gammelmark, M Nielsen, T Rix, H Vadmann, K Andersen, B Christensen, L Hessing Kobbelgaard, B Mikkelsen, M Storgaard; *Esbjerg Hospital:* O Nyvad, A Rohold, K Thomsen, J Hummelshoj, A Svenningsen, L Tanggaard; *Gentofte Sygehus, Hellerup:* G Gislason, C Torp Pedersen, G von Jessen, J Larsen, J Sandberg Madsen; *Glostrup Hospital:* H Iversen, C Nielsen, J Obionu, S Simonsen, M Kjaergaard Danö, P Hornslet, T Veng-Olsen; *Herning Hospital:* O May, L Madsen, M Engbjerg Andersen, L Jensen, V Lynggaard, S Søndergaard, S Vester; *Holbæk Hospital:* N Roseva-Nielsen, V Sørensen, K Skjødeberg Christensen, M Bang Hansen, L Jensen, H Mollerup, S Voigt; *Kolding Hospital:* J Jepsen, J Gesla, L Johansen, E Zeuthen, B Bjerge Kaspersen, B Felthaus, M Løkke, L Holm Pedersen, A Schrader, L Schmidt Thomsen; *Kolesterollaboratoriet, Gentofte:* S Stender, T Brink-Kjaer, H Jonsson; *Næstved Hospital:* R Sykulski, J Thorsen, P Osterby Elin, B Stage Jensen; *Nordsjællands Hospital, Helsingor:* N Ralfkier, H Gottschalck, S Bloksgaard Nilesen; *Odense Universitets Hospital:* M Lytken Larsen, H Mickley, S Hosbond, L Saaby, M Ronn, I Rosenlund; *Rigshospitalet, Copenhagen:* P Clemmensen, P Grande, L Køber, H Andersson, S Wiberg, K Graversen, L Hedgaard, M Tarras Wahlberg; *Roskilde Hospital:* T Melchior, C Larsen, S Heinsvig, I Larsen, V Perret-Gentil; *Silkeborg Hospital:* L Frost, A Christensen, H Arp, M Mortensen, A Odgaard; *Skejby Hospital, Århus:* H Wiggers, S Poulsen, K Serup-Hansen, G Udsen; *Slagelse Hospital:* J Lomholt, H Mollerup, K Niemann; *Steno Diabetes Center, Gentofte:* M Ridderstråle, L Tarnow, T Boesgaard, T Hansen, N Safai, M Andersen, S Hansen, M Pedersen; *Svendborg Hospital:* K Egstrup, S Auscher, A Osmanagic, H Sheta, K Vinter, L Hindsgaul, M Lundgaard, L Moltrup; *Viborg Hospital:* I Klausen, B Haastrup, B Hedegaard, S Gudmundsdottir;

Finland
National coordinator: A Kesäniemi; *Collaborators: Etelä-Karjalan Keskussairaala, Lappeenranta:* E Hussi, S Valpas; *FinnMedi Oy, Tampere:* J Taurio, A Airaksinen, S Luukkonen, S Uusitalo; *Geri-Med Oy, Helsinki:* T Strandberg, E Ronkainen, C Sarti, R Tilvis, M Aaltonen, E Landström, A Punkka; *Kuopion Liikuntalääketieteen Tutkimuslaitos, Kuopio:* T Lakka, K Savonen, H Kastarinen, N Koskinen; *Kuusankosken Terveysasema, Kuusankoski:* M-L Tuominen, A Haaraoja; *Lapin Keskussairaala, Rovaniemi:* J Laukkanen, A Hadjikov, S

Hellsten, P Hiltunen; *Menoa Oy, Kinkomaa:* M Perhonen, M Valtonen, M Moilanen, M Varakas; *OYS, Oulu:* A Kesäniemi, E Eloranta, O Ukkola, P, Ojala, L Ukkola; *Pohjois-Karjala Projektisäätiö, Joensuu:* S Pihlman, M Mononen, K Hyttinen, S Lipponen; *Seinäjoen Lääkäritalo, Seinäjoki:* M Kotila, A Pöllänen, A Rajala; *Turun Yliopistollinen Sairaala, Turku:* I Kantola, T Kiviniemi, M Strandberg, J Raali, E Roine;

Germany

National Coordinator: C Wanner; *Collaborators: Ambulantes Herzzentrum, Kassel:* K-F Appel, S Appel, A Utech, P Becker, S Chmilewski, J Kuehnert, I Pietsch, A Reinemann, S Werner; *Cardiopraxis, Mainz:* G Mentz, M Drexler, I Müller-Wittlich, A Drexler, S Hobrack, K Tajouaout; *Charité Campus Virchow-Klinikum, Berlin:* H-D Düngen, T Bekfani, G Cherian, M Fritschka, L Musial-Bright, T Trippel, V Tscholl, A Baltic, S Inkrot, A Maiwald, A Pinta, M Sacirovic, Y Saewe, R Stolz; *Comprehensive Heart Failure Center [CHFC] and Department of Internal Medicine I, University Hospital, Würzburg:* CE Angermann, G Ertl, S Brenner, K Boelmans, M Breunig, F Hammer, U Hofmann, B Meyer, C Wanner, J Judex, A Knoppe, C Richter; *DRK Kliniken, Berlin:* H Voehringer, E Lianopoulos, C Opitz, M Buchholz, S Gebhardt, S Helms; *Forschungszentrum Ruhr, Witten:* T Horacek, G Kahrmann, O Stobbe, P Fink, A Günesli, J Richtstein, K Wilke; *Gefäß Zentrum Universitaetsklinik, Dresden:* N Weiss, N Jabs, A Mahlmann, S Werth, S Brilloff, M Dechert, E Festerling, M Leistner, B Sehr, I Weise; *Gemeinschaftspraxis Dr. Haggenmiller und Dr. Jeserich, Nuernberg:* M Jeserich, S Haggenmiller, S Kimmel, H-O Schoengart, M Cakir, G Eichinger, M Rupprecht; *Juedisches Krankenhaus, Berlin:* K Graf, R Thieme, E Tummos, J Ausner, L Fischer; *Kardiologie Universitaet, Magdeburg:* R Braun-Dullaeus, H Bönigk, S Meißler, A Schmeißer, H Schulz, S Uslar, D Weigt, R Gebauer, S Roeder, K Schäfer; *Kardiologische Geimeinschaftspraxis, München:* S Silber, M Basler, C Matt, P Styllou, B Bosnjak, M Huth, A Schmid, C Senger; *Kardiologische Gemeinschaftspraxis, Würzburg:* M Camerer, H Drösch, H Strömer, J Heid, R Wilsch; *Klinik am See, Ruedersdorf:* H Völler, A Jawari, S Stiehl, A Salzwedel, K Stolze; *Kerkhoff Klinik, Bad Nauheim:* V Mitrovic, L Gaede, A Peil, M Shaker; *Klinikum Bielefeld:* C Stellbrink, C Drephal, B Elberg, J Junge, E Stellbrink, T Weber, B Brettschneider, C Gruhne, M Iselt, J Kube, U Lehmann, C Potthast, S Watson; *Klinikum Coburg:* J Brachmann, M Held, C Mahnkopf, A Saleh, A Sallam, B Schertel-Gruenler, S Schnupp, U Goebel, S Rube, K Truthan; *Klinikum Frankfurt Hoechst:* C Kadel, K Lahiri, H Moellinger, M Pagitz, J Reusch, A Stadler, N Zulauf, V Anuschek, S Beißner, M Buerger, M Hagmanns, E Hickmann, C Klinger, G Rahn, J Schemann, E Tu, C Wölfl; *Klinikum Fulda:* V Schaechinger, T Pluecker, G Strupp, T Trepels, W Wahl, C Roemmelt, S Fritsch; *Klinikum Leverkusen:* P Schwimmbeck, A Fahrig, M Hautmann, A Öner, B Weidmann, I Wenzel; *Klinikum Universität München - Campus Innenstadt:* U Hofmann, M Czihal, K Hausleiter, K Kress, R Kreuzpointner, P Kuhlencordt, S Rieber, J Nuerbchen, S Roth-Zetzsche; *Klinikum Universität München - Grosshadern:* S Kääb, W Franz, C Feldmann, U Grabmaier, M Sinner, A Bongartz, C Gross, B Halter, J Sakic; *Medizinische Hochschule, Hannover:* J Bauersachs, U Bavendiek, J Pirr, K Sonnenschein, K Hohenleitner-Lührßen, A Juergens, N Schaefer; *Praxis fuer Kardiologie Dr. Bosiljanoff, München:* P Bosiljanoff, G Betzl, E Bosiljanoff, J Feger, A Kinateder; *Robert Bosch Krankenhaus, Stuttgart:* U Sechtem, S Egenrieder, A Karagianni, T Schäufele, M Voehringer, S Gruensfelder, L Hoffmann, I Wenzelburger; *SLK-Kliniken Heilbronn, Bad Friedrichshall:* T Dengler, C Loges, C Neatu, C Lindner, C Pfau; *St. Johannes Hospital, Dortmund:* H Heuer, H Bourhaial, I Dulea, B Elberg, O Guerocak, S Halberstadt, E Kemala, J Peterek, H Philips, U Dieckheuer, K Euler, B Laschewski, M Maas, J Peda; *Studienzentrum Prof. Hanefeld, Dresden:* F Schaper, E

Henkel, M Teige, C Schrapel, K Waetzold; *Universität Magdeburg Lipidambulanz:* B Isermann, S Westphal, K Borucki, M Schulze, K West; *Universitäts-Herzzentrum Freiburg-Bad Krozingen:* D Trenk, W Hochholzer, S Leggewie, C Stratz, A Schiebeling-Romer, M Siefer; *Universitätsklinikum, Bonn:* N Werner, R Schueler, A Sedaghat, J-M Sinning, K Twelker, U Jones, M Lauterborn, M Lennarz, S Lubnau, A Meier, T Streuber-Bouhksas; *Universitätsklinikum, Hamburg:* S Blankenberg, M Adam, C Alternburg, M Huemmelgen, A Jagodzinski, M Karaks, K Koopmann, S Schäfer, H Schüler, K Sydow, C Thomas, E Tigges, I De Boer, M Hermes, J Nebel, C Schlesner, J Schlüter, D Sprechert, S Zbik; *Universitätsklinikum Münster Kardiologie:* J Waltenberger, D Fisscher, C Schulz; *Universitätsklinikum Münster Lipidambulanz:* B Otte, L Centofante, R Kremerskothen, J Beilker, S Müller, E Schlosser; *Universitätsklinikum Regensburg:* C Birner, A Luchner, J Egresits, C Jungbauer, M Resch, P Schmid, M Buesing, C Liebl, S Sülflow; *Universitätsklinikum Schleswig-Holstein, Luebeck:* J Weil, B Brueggemann, T Graf, C Moeller, M Miodek; *Vivantes Klinikum am Urban, Berlin:* H Ince, D Andresen, A Seidel, C Sprenger, Y Stoeckicht, S Ziefle, S Forster, J Ort, S Szczesnak;

Italy

National coordinator: AP Maggioni; Collaborators: *Albano Laziale, Ospedali Riuniti Albano-Genzano:* P Midi, A Felici, F Caranzetti, M Miccoli, L Tomassini; *Aosta, Ospedale Generale Regionale-PO U.* Parini: M Sicuro, C Aillon, C Gianonatti, C Baré, P Donà; Bari, Ospedale San Paolo: P Caldarola, M Resta, M Ruggiero, M Galietti; *Bologna, Ospedale Maggiore:* G Di Pasquale, E Filippini, L Riva, S Zagnoni; *Casarano, Presidio Ospedaliero F. Ferrari:* G Piccinni, C Perrone, A Aloisi; *Cortona, Ospedale Valdichiana Santa Margherita:* F Cosmi, B Mariottoni, B Tarquini; *Cremona, Ospedale di Cremona:* S Frattini, S Pirelli, G Paradiso, S Signore; *Firenze, Ospedale San Giovanni di Dio:* C Baggiore, C Crescenti, A Leopardi, S Benoni, P Ferruzzi, P Pini; *Foggia, Ospedali Riuniti:* M Di Biase, C D'Antuono, R Ieva, I Monaco, D Montrone, S Musio; *Gubbio, Ospedale Gubbio-Gualdo Tadino:* S Mandorla, M Buccolieri, E Capponi, S Martinelli, N Piccioni, O Regni, A Iaquaniello, A Malvestiti, S Pieroni Minciaroli; *Isernia, Ospedale F. Veneziale:* C Olivieri, R Chiodi, A Masciotra; *Legnano, Ospedale Civile:* F Poletti, S De Servi, S Affinito, A Di Donato, S Messina, C Stefanin; *Lido di Camaiore, Nuovo Ospedale Versilia:* G Casolo, L Robiglio, F Vivaldi, A Buono, C Urbani; *Milano, Ospedale Niguarda:* A Alberti, E Giagnoni, T Pupilella, A Biondi, A Di Donato, A Lazzari, S Messina; *Orbassano, Ospedale San Luigi Gonzaga:* L Montagna, C Chirio, I Salvetti, M Perrelli; *Palmanova, Ospedale di Palmanova:* MG Baldin, R Cesanelli, S Boccati, G Durì; *Pavia, IRCCS -Fondazione Salvatore Maugeri:* S Priori, M Ceresa, M Zambelli, A Biondi, A Di Donato, S Messina, G Savino; *Rimini, Ospedale Infermi:* G Piovaccari, D Grosseto, P Testa, P Gaviani, A Girardi, I Manzo, G Serroni; *San Felice a Cancello, Ospedale Ave Gratia Plena:* C De Matteis, U Campidonico, C Crisci, M Falco, C Di Matteo, I Manzo; *Santa Maria Capua Vetere, Ospedale San Giuseppe e Melorio:* L Fattore, G Morello, C Nave, C Di Matteo, I Manzo; *Sarzana, Ospedale San Bartolomeo:* R Petacchi, D Bertoli, G Filorizzo, A Buono, C Urbani; *Scorrano, Ospedale Ignazio Veris Delli Ponti:* O De Donno, E De Lorenzi, L Urso, A Aloisi, A Lecci; *Sondrio, Ospedale Civile:* G Cucchi, E Gianatti; *Terni, Azienda USL Umbria 2:* G Proietti, M Bernardinangeli, G Proietti, S Serani; *Udine, AOU Santa Maria della Misericordia:* G Morocutti, T Bisceglia, C Fresco, V Andrioli, V Biundo; *Veruno, Fondazione Salvatore Maugeri:* P Giannuzzi (deceased), M Gattone, V Bolzani, M Di Ruocco, A Biondi, A Di Donato, S Messina, B Temporelli;

Japan

National coordinators: S Goto, T Teramoto; Collaborators: *Institute of Brain and Blood Vessels Mihara Hospital:* B Mihara; *National Hospital Organization Kyushu Medical Center:* Y Okada; [Note: These collaborators participated in preparations to initiate recruitment in Japan, but it was subsequently planned not to extend recruitment there.]

Norway

National coordinator: O Nygard; *Collaborators: Førde sentralsjukehus, Førde:* T Vingsnes, H Sirnes, K Solheim, R Tilseth, M Vestre, A Bjorkas, I Vassbotten; *Haugesund Hjertepraksis, Haugesund:* R Rød, R Stodle; *Haukeland Universitetssykehus, Bergen:* O Nygård, C Berge, H Schartum Hansen, A Kask, K Løland, G Svingen, N Tuseth, V Vavik, E Wilberg Rebnord, B Gjellefall, S Hovland, S Nordgaard Thorsen; *MEDI3 Ålesund, Ålesund:* T Kjærnli, O Erstad, K Grödal, S Nybø, C Royset, S Stadsnes; *Nordland Hjertesenter AS, Bodø:* A Hovland, K Lappegard, J Sandvik, H Carlsen, T Enebakk, H Thunhaug; *Norsk Helseklinikk, Lierskogen:* L Solnør, P Holmstrom; *Skedsmo Medisinske Senter AS, Skedsmokorset:* K Risberg, H Hansen; *St. Olavs Hospital, Trondheim:* B Kulseng, K Lauglo, H Tevik Bjøru, T Langeng, S Salater; *Stavanger Helseforskning AS, Stavanger:* P Scott Munk, E Singsaas, A-G Larsen, S Moen, J Nilsen; *Sykehuset Innlandet HF, Hamar:* K Andersen, T Larsen, E Turkerud Söby; *Sykehuset Innlandet HF, Kongsvinger:* J Sparby, E Werenskjold; *Sykehuset Innlandet HF, Lillehammer:* M Grundtvig, M German, G Szacinski; *Sykehuset Telemark, Skien:* J Hysing, J Thalamus, E Flagstad, H Rosland; *Ullevål Universitetssykehus HF, Oslo;* T Pedersen, T Klemsdal, L Bergengen, R Kleve; *UNN Hjerte/Kar Poliklinikk, Tromsö:* A Skogsholm, K Larsby, I Holde, R Jonassen, M Nilsen; *Vestfold Hjertesenter AS, Sandefjord:* J Berg-Johansen, H Tisthammer Antonsen;

Sweden

National coordinator: L Jonasson; *Collaborators: A+ Science, Stockholm:* Å Ohlsson, L Bastani, T Delgado, S Gunvarsdotter, P Löf, L Persson, T Larsdotter-Damm, K Skoglund; *Capio Lund, Lund:* C Lindholm, J Thulin, E Assarsson, M Broberg; *Centralsjukhuset Kristianstad, Kristianstad*: I Torstensson, I Lager, K Hårsmar, A Knutsson; *City Heart, Stockholm:* L Hjelmaeus, R Zlatewa, E Lindemann, I McLain; *Dalecarlia Clinical RC, Rättvik:* H Larnefeldt, M Eld, M Björkman-Larnefeldt; *Hallands Sjukhus, Halmstad:* P Hårdhammar, P-A Johansson, A-C Karlsson, M Lingman, M Löfgren, A Tabandeh, L Andersson, C Palm; *Hässleholms Sjukhus, Hässleholm:* I Timberg, M Stjernberg, P Wikström; *Karolinska Universitetssjukhuset, Stockholm:* C Bergmark, O Thott, U Hedin, C Montan, O Nilsson, M Lenquist; *Linköpings Universitetssjukhus, Linköping:* L Jonasson, L Nilsson, P Wodlin, M Börjesson, A Raschberger; *Ljungby Lasarett, Ljungby:* L Ekholm, K-A Svensson, A Ågårdh, L Algotsson, M-L Bergström; *Universitetssjukhuset Mölndal, Mölndal:* F Randers, L Klintberg, U Axelsson, P-Å Moström, G Mourtzinis, P Parén, B Persson, M Risenfors, J Moodh, M Mossmark, L Ohrtgren; *Motala Lasarett, Motala:* P Ahlström, Å Törnqvist, U Rosenqvist, M Grändås, G Karlsson; *Norrlands Universitetssjukhus, Umeå:* P Ottander, A Eriksson, M Backlund, M Johansson, C Sundholm; *Öbackakliniken, Härnösand:* A Kempe, S Salomonsson, J Larsson, H Andersson, K Forsberg, A Sjödin; *Oskarshamns Sjukhus, Oskarshamn:* U Mathiesen, M Carlsson, E Keppel, K Fehling, U Robertsson; *Skellefteå Lasarett, Skellefteå:* J-H Jansson, K Boman, M Johansson, L-M Lundmark, B Norrfors; *Universitetssjukhuset, Örebro:* A Weiderman, E Jasinska, M Lundvall, K Eriksson, J Kjellberg-Eriksson, U Larsson; *Växjö Sjukhus, Växjö:* P Vasko, G Anderson, O Bergström, S Johansson, T Nyström, I Uggeldahl;

United Kingdom

National coordinator: L Bowman; *Collaborators: Aberdeen Royal Infirmary, Aberdeen:* J Webster, J Henderson, V Herd, E Wilson; *Addenbrooke's Hospital, Cambridge:* F Mir, S Blackwood (monitor), M Watts; *Barnsley District General Hospital, Barnsley:* W Khan, N Tahir, K Elliot, J Lichfield, H Marsh, M Reid; *Bedford Hospital, Bedford:* I Cooper, R de Silva, A Gallivan; *Birmingham Heartlands Hospital, Birmingham:* A Jones, L Andrews, C Jewkes; *Bradford Royal Infirmary, Bradford:* S Lindsay, K Rees, A Wilson (monitor); *Bristol Royal Infirmary, Bristol:* G Bayly, J Chambers (monitor), S George, M Halestrap; *Colwyn Bay Community Hospital, Colwyn Bay:* C Bellamy, S Evans, J James, E Pritchard, J Stockport, S Wynne; *Derriford Hospital, Plymouth:* J Fulton, J Simmonds, C Stewart, C West; *Dewsbury District Hospital, West Yorkshire:* H Chidambara, T Msimanga, B Moore, G Roberts; *Dorset County Hospital, Dorchester:* T Edwards, S Breakspear, N Fleming; *Edinburgh Royal Infirmary, Edinburgh:* D Newby, E Fraser, L Marshall, H Nailon; *Guy's Hospital, London:* J Chambers, D Parkin; *Hillingdon Hospital, Uxbridge:* M Edwards, C Mitchell, N Mahabir; *Huddersfield Royal Infirmary, Huddersfield:* H Griffiths, K Mitchell, D Appleyard, S Farr; *King's Mill Hospital, Sutton-in-Ashfield:* R Lloyd-Mostyn, S Hardingham, T Sewell; *Lister Hospital, Stevenage:* M Lynch, W Burog, M Dhaliwal, C Mfuko; *Luton and Dunstable Hospital, Luton:* C Travill, S Gent, B Norris; *Macclesfield Hospital, Macclesfield:* R Edgell, T Lake, A Taylor Bennett (monitor); *Manor Hospital, Walsall:* A Hartland, E Walton (monitor); *Memorial Hospital, Darlington:* J Murphy, G Brennan, P Cawley, L Dixon, E Rees; *Musgrove Park Hospital, Taunton:* R Andrews, T Brownlow, S Crouch, H Mills, M Nixon, N Salter; *Ninewells Hospital, Dundee:* S Pringle, S Hutcheon, H Waldie; *North Manchester General Hospital, Manchester:* J Swan, D McSorland; *North Tyneside General Hospital, North Shields:* R Curless, M Armstrong, C Ashbrook-Raby, D Bunn, R Gour, C Herriott, J James, C Robson, C Tanney, A Taylor Bennett (monitor); *Northampton General Hospital, Northampton:* P Davey, L Campey, K Smith, E Tanqueray; *Pinderfields Hospital, Wakefield:* A Munir, O Pereira, M Khalifa, B Moore; *Princess Royal Hospital, Telford:* N Capps, D Donaldson (monitor), C Miller, L Tonks; *Queen's Hospital, Burton-upon-Trent:* T Reynolds, P Basvi, J Reynolds, L Wilcox; *Queen's Medical Centre, Nottingham:* P Mansell, G Babington, E Barnes, S Beck, S Craig, L Patterson, A Selby, C Woodford; *Richard Doll Building, Oxford:* L Bowman, MJ Landray, J Armitage, H Watkins, S Beebe, K Bowsher Brown, J James, H Lochhead, J Robertson, V Toghill, L Wright, L Young; *Rotherham District General Hospital, Rotherham:* R Muthusamy, M Lawan, C Weston; *Royal Berkshire Hospital, Reading:* W Orr, J Foxton, S Hallett, P Hilltout, L Jones, J King; *Royal Blackburn Hospital, Blackburn:* S Ramtoola, Y Grimes; *Royal Bolton Hospital, Bolton:* A Hutchesson, J Cummings, K Morris; *Royal Cornwall Hospital, Truro:* S Fleming, K Ludlow, M Parrett, S Pellow, L Quinn; *Royal Devon & Exeter Hospital, Exeter:* M James, E Green, S Keenan; *Royal United Hospital, Bath:* J Reckless, A Robinson, G Andrews, A McLenaghan; *Royal Victoria Hospital, Newcastle upon Tyne:* I ul Haq, C Albers; *Russells Hall Hospital, Dudley:* M Labib, E Higginson; *Salford Royal Hospital, Salford;* A Fitchet, E Darrel-Asherel, J Green, M Healey, K Morris, D Sexton; *Sandwell General Hospital, West Bromwich:* E Hughes, J Chackathayil, S Willetts; *Southampton General Hospital, Southampton:* C Shearman, N Pal, A Lewis, M Pasinabo, C Trevithick, D Tyler, B Watkins; *Southmead Hospital, Bristol:* M Papouchado, G Andrews, W Bertram, E Binley, S Hierons, S Kandola, C Mann, K Whitney; *St Helier Hospital, Carshalton:* H Wilcox, A Bibi, J Fuller, S Jackson; *St Mary's Hospital, Portsmouth:* P Kalra, S Howe, K Hudson, A Suttling, C Turner; *St Richard's Hospital, Chichester:* Y Wong, L Clayton-Evans, S Moore, S Stearn; *Stepping Hill Hospital, Stockport:* P Lewis, H Cochrane, J Curtis, M Holland; *Sunderland Royal Hospital, Sunderland:* S Junejo, E

Dungca, T Robson, A Smith, A Taylor Bennett (monitor); *Torbay Hospital, Torquay:* C Carey, L Felmeden, A Summerhayes, J Sutton; *University Hospital of North Staffordshire, Stoke-on-Trent:* R Butler, J Creamer, J Bellaby, K Castro-Foskett, M Griffiths, J Machin, I Massey, E Sellars, J Wain*; University Hospital of Wales, Cardiff:* I McDowell, L Davies, M Davies, H Dyer, M Odam, A Waters; *University Hospital, Coventry:* M Been, V Ansell, A Campbell, D Davies, B De Burca, J Jones, A Musa; *Victoria Hospital, Blackpool:* D Roberts, R Brady, C Dickinson, L Lane, S Pickervance; *Victoria Hospital, Kirkcaldy:* M Francis, V Bryson; *Watford General Hospital, Watford:* M Clements, L Ashton, A George, K Markwell, E Walker; *West Cumberland Hospital, Whitehaven:* O Orugun, U Poultney; *Whipps Cross University Hospital, London:* F Lie, A Taneja, B Badal, V Conteh, M Jones, M Montemayor; *Worcestershire Royal Hospital, Worcester:* J Trevelyan, E Byng-Hollander, A Doughty; *Worthing Hospital, Worthing:* M Signy, A Dunne, H Fox, S Moore, S Stearn, K Wheatley; *Wycombe General Hospital, Wycombe:* S Price, N Mahabir; *Wythenshawe Hospital, Manchester:* S Ray, S Golledge, M Holland, M Murmu, A Nicholas; *Yeovil District Hospital, Yeovil:* G Brigden, J Board, C Buckley, C Vickers;

United States of America

National coordinator: CP Cannon; *Collaborators: Acadia Clinical Research LLC, Bangor, ME:* M Albert, G Baillargeon, D Harman; *Advanced Heart Care, LLC, Bridgewater, NJ:* S Mahal, J Kaur, S Padkowsky, M Walker, S Yandamuri; *Advanced Neurology Specialists, Great Falls, MT:* D Dietrich, L Armstrong, R Brown, M Casey, V Schaefer; *Albuquerque Clinical Trials, Inc., Albuquerque NM:* E Bretton, D Hsi, J Kovach, J Troy; *Asheville Cardiology Associates, Asheville, NC:* B Asbill, L Brown, T Cauthren, A Hull, O Lim, J Tompkins, J Vaughn; *Associated Cardiovascular Consultants, Voorhees, NJ:* R Perlman, D Connors, D Hoopes, D Palazzo, A Prosser, M Serrano-Rawls; *Associated Research Partners LLC, Jonesboro, AR:* B Tedder, E Johnson, T Pearson, K Rubino, P Williams; *Atlanta Heart Specialists, LLC, Cumming, GA:* N Singh, M Brown, S Dubal, E Hall, D Logwood, U Mazahir, K Raynes; *Austin Heart, PLLC, Austin, TX:* R Gammon, A Bauman, J Hatch, P Mock, N Tilton; *Austin Heart, PLLC, Jonesboro, AR:* W Abide, Jr., D Gudeman, S Minor, T Shipwash; *Aventura Heart Center, Aventura, FL:* D Korn, A Korn; *Awasty Research Network, LLC, Marion, OH:* V Awasty, E Baldwin, G Hunt, V Kaiser, C McMurray; *Bay Area Cardiology Associates P.A, Brandon, FL:* T Khan, J Al-Jumaily, T Foster, V Holbrook; *Baylor College of Medicine, Houston, TX:* V Nambi, C Ballantyne, M Jackson, P Jones, B Morris, M Techmanski, A Tran; *Beverly Hills Cardiology, Los Angeles, CA:* S Eshaghian, H Mirshkarlo; *Black Hills Cardiovascular Research, Rapid City, SD:* A Zineldine, J Bies, D Hockett, L Kimball; *Boice Willis Clinic, Rocky Mount, NC:* M Thakkar, S Varma, S Barkley-Daughtry, S Collins, S Evans-Gay, L Martin, A McKinley, L Murray, L Noel, S Prasada, R Robinson, S Wheeler; *Brigham and Women's Hospital, Boston, MA:* CP Cannon, J Andreo, S Bansilal, B Bergmark, E Bohula May, M Cavender, J Cyr, N Desai, C Fanola, N Fantony, R Giugliano, J Gutierrez, P Kazanjian, J Marti-Bernier, J Mega, R Mesa, M O'Donoghue, B Scirica, M Silverman, D Steen, L Williams; *Bryan Heart, Lincoln, NE:* C Meckel, C Orosco, R Saalfeld, N Thompson, C Wiechert; *Buffalo Medical Group, P.C., Williamsville, NY:* L Kozlowski, B Cooke, J Corbelli, A Galla, R Stock; *Capital Cardiology Associates, Troy, NY:* R Benton, A Carroll, C Leeper, E Orvis; *Capital Cardiology Associates, Albany, NY:* P Shah, A Kasson, J Lieberman, A O'Malley, E Orvis; *Cardiology Associates of Bellin Health, Green Bay, WI:* J Rider, B Loomis, M Schantz; *Cardiology Associates of Fairfield County, P.C., Stamford, CT:* M Heiman, K Sadowski, L Scierka, J Sclafani, K Strubberg, D Mania, E Del Mastro; *Cardiology Associates of Fairfield County, P.C., Trumbull, CT:* R Jumper, K Bukoski, P

Eiben, R Keegan, E Kelley, E Sekerak, J Weisberger, A Serra; *Cardiology Associates of Fairfield County, P.C., Norwalk, CT:* C Augenbraun, S Jumper, A Stuart, A Archer, T Malak, J Velky; *Cardiology Consultants, Pensacola, FL:* R Spencer, B Lane, J Lehmann; *Cardiology Research Associates, Daytona Beach, FL:* D Henderson, L Crandall, A Easterling, A Lizama, D Millard; *Cardiovascular Associates of the Delaware Valley, PA, Elmer, NJ:* M Gelernt, C Billings, D Cockrell, E Anderson; *Cardiovascular Associates of the Delaware Valley, PA, Haddon Heights, NJ:* A Pavlides, M Davis; *Cardiovascular Associates of the Delaware Valley, PA, Sewell, NJ:* D Viswanath, M Kinder, H Jeffers, S Manga, P Shaw; *Cardiovascular Associates of the Southeast, Birmingham, AL:* S Jones, T Stover; *Cardiovascular Associates of the Southeast, Birmingham, AL:* R Reeves, S Frew; *Cardiovascular Institute of the South, Opelousas, LA:* R Menuet II, K Veerina, N Domingue, L Huffman, Y Leach, T Rideaux, J Smith, L Soileau; *Cardiovascular Research Foundation of Southern California, Beverly Hills, CA:* R Karlsberg, Bhatia, A Gomez, L Levi, D Lopez; *Cardiovascular Research of Knoxville, Knoxville, TN:* C Treasure II, L Michaelis, M Parker, C Robertson, L Treasure; *CentraCare Heart and Vascular Center at St. Cloud Hospital, St. Cloud, MN:* B Erickson, A Amundson, J Humbert, H Madden; *Charles River Medical Associates, Natick, MA:* V Desai, K Lemmertz, J Zoghbi; *Charlotte Heart Group Research Center, Port Charlotte, FL:* M Malone, K Mullinax, R Schenks; *Chesapeake Cardiovascular Associates, Baltimore, MD:* D Goldscher, M Fisher, J Latteri; *Chesapeake Cardiovascular Associates, Towson, MD:* M Goldstein, H Lutz; *Chesapeake Cardiovascular Associates, Baltimore, MD:* D Peichert, E Haskel, J Powell, C Yashinski; *Clearwater Cardiovascular and Interventional Consultants, Clearwater, FL:* J Amin, D Bashton, S Burns, A Davidson, C DeSousa, C Humberger, R McGee; *Clearwater Cardiovascular and Interventional Consultants, Safety Harbor, FL:* J Zelenka, D Ferguson, C Manuel, J Quinn, J Zelik; *Cleveland Clinic Fairview Cardiovascular Medicine, Fairview Park, OH:* E Nukta, B Bittel, M Dettmer, C Palmer; *Clinical Research Associates, Florence, SC:* W Boulware, L Cooper, R Freeman; *Clinical Trials of America, Shreveport, LA:* W Zhang, K Banks, L Hall, C Hall, K Riser, S Vaz, J Winstead, L Womack; *Clinical Trials of America, Inc, Lenoir, NC:* J Dy, L Fox, E Landers, B Raby, T Whisnant; *Clinical Trials of America, INC, Hickory, NC:* S Isserman, T Annas, K Kirby, J Lail, C Moore, A Waters; *Cohen Medical Associates, Delray Beach, FL:* R Cohen, J Bossaers, L Heaney, A Hislop, L Moreiras, M Ocampo; *Community Clinical Research Center, Anderson, IN:* P Jetty, T Allen, C Custer, S Howard, A Key, S Lipps; *Comprehensive Cardiovascular Medical Group, Bakersfield, CA:* S Banerjee, S Carlos, A Garza, R Sutton; *Dayton Heart Center, Dayton, OH:* J Tobiansky, J Gluck, C Tofstad; *Doylestown Health Cardiology, a Division of Doylestown Health Physicians, Doylestown, PA:* J Kmetzo, J Brown, L Carter, R Riley, D Scott, P Seger, D Taylor, D Wood; *East Texas Cardiology, PA, Houston, TX:* A Ahmad, M Ahmed, H Ayub, S Contreras, S Iqbal, S Martinez, M Martinez; *Eastern Suffolk Cardiology, Southampton, NY:* S Donahoe, P Dalal, C Defraia, R DeStefano, S Lederman, D Lorme, M Ruhani; *Escondido Cardiology Associates, Inc, Escondido, CA:* R Acheatel, J Biggers, P Emery; *Florida Hospital, Orlando, FL:* C Kim, D Barnes, K Behm, A Dziekonski, H Karunaratne, C Stastny; *Gemini Scientific, LLC, Madison, WI:* N Bittar, S Lehmann, M Spatola, P Wilson; *Gotham Cardiovascular Research, New York, NY:* C Staniloae, E Homberg-Pinassi; *Grand View-Lehigh Valley Health Services, Buxmont Cardiology Division, Sellersville, PA:* P Hermany, K Batchlett, A Gibson, S Meissner-Dengler; *Green and Seidner Family Practice Associates, Lansdale, PA:* J Rosenfeld, B Madden, M Seidner, K Sosonkin; *Harrisonburg Medical Associates, Harrisonburg, VA:* S Pollock, S Johnson; *Health First Medical Group, Melbourne, FL:* J Salazar, R Hovland, J Jordan, S Karas, T Peacock, N Schechtmann, G Tischner, R Vicari, K Warren; *Heart and Health Institute Westside, Plantation, FL:* A Ghitis, H

Cusner, M Klaus Clark; *Heart Center at St. Mark's Hospital, Salt Lake City, UT:* J Zebrack, S Christensen, C Evenson, D Fullerton; *Heart Center Research LLC, Huntsville, AL:* J Hartley, K Broadway, L Eskridge, D Raymond; *HeartCare Midwest, Peoria, IL:* T Kizhakekuttu, S Hillis, R Klundt, D McElroy; *Heritage Valley Medical Group, Inc, Beaver, PA.:* K House, R Begg, J Acon, A Flores, J Hobbs-Williams, E Schidemantle; *HOPE Research Institute, Phoenix, AZ:* M Cooper, E Campbell, B Corcoran, S Hughes, N Miller, S Steingard; *HOPE Research Institute, Chandler, AZ:* D Einhorn, M Berry, S Dawkins Hughes, L Gilbert, E Lasala, A Loeck, N Mills, J Oppenheim; *Hudson Valley Cardiovascular Practice, P.C., Poughkeepsie, NY:* D O'Dea, S Brian, G Gerber, T Landi, J Ling, S Rimmey; *Imperial Health, LLP, Lake Charles, LA:* R Gilmore, C Bruney, E Gabbert, R Hays, L Stawecki, J Trahan, D Winey-Ward; *Indian River Medical Center – Cardiology, Vero Beach, FL:* S Baker, B Gervasio, J Labodin; *Inova Cardiology Ambulatory Research, Manassas, VA:* H Taheri, J Brooks, A Delozier, J Jayashekaramurthy, S Khachab, P Machineni, K Morgan; *Intermed, PA, Portland, ME:* C Cathcart, E Ciampanelli, W Ervin, K Soule, J Stinson; *Iowa Diabetes and Endocrinology Research Center, Des Moines, IA:* A Bhargava, L Borg, A Carver; *Jacksonville Center for Clinical Research, Jacksonville, FL:* M Koren, A West; *Kootenai Heart Clinics, LLC, Coeur d'Alene, ID:* R Jenkins, S Barnett, H Caro, J Mooney; *Kootenai Heart Clinics, LLC, Spokane, WA:* M Janout, J Bjergo, E Kelley, L Passey, K Sather; *Kore CV Research, Jackson, TN:* E Hage-Korban, M Carrington, A Childs, A Harrington, D Manns, T Phelan; *LeBauer Cardiovascular Research Foundation, Greensboro, NC:* T Stuckey, S Lord, S Milks; *Louisville Metabolic and Atherosclerosis Research Center, Louisville, KY:* H Bays, D Bushong, S Keiran, M Moore, K Weiter; *Lutherville Personal Physicians, Lutherville, MD:* F Morris, C Dignon, J Downing, D Lowry, A Metcalf; *Maine Research Associates, Auburn, ME:* E Claxton Jr., R Weiss, S Dumais; *Marin Endocrine Care & Research, Inc, Greenbrae, CA:* R Bernstein, C Singh; *McLaren Northern Michigan, Petoskey, MI:* H Colfer, A Teklinski, D Antonishen, M Antonishen, M Ronquist, C Shaw; *Medicor Cardiology, Bridgewater, NJ:* J Hall, C Hanzich; Meriter Hospital, Inc., Meriter, Madison, WI: D Lewis, A Gessler, L Skatrud; *Michigan Cardiovascular Institute, Saginaw, MI:* J Collins, V Bitzer, A Fruge, T Gauthier, M Hernandez, K Kayner, C Michon, L Naessens; *MidMichigan Medical Center Midland, Midland, MI:* W Felten, A Cryderman, M Lagalo, K Mostek, C Cluley, J Prior; *MidValley Cardiology, Kingston, NY:* E Lader, M Meyer; *Mobile Heart Specialists, PC, Mobile, AL:* C Alford, S Bryan, M Craig, J Gilley; *MODEL Clinical Research, Baltimore, MD:* P Levin, L Bromberger, D Lowry; *MultiCare Institute for Research & Innovation, Tacoma, WA:* D Guerra, P Brandon, C Burton, J Ebert, K Garrison, C Goetz, S Harris, C Lumsden, D Quinn; *MultiCare Research Institute, Tacoma, WA:* R Graf, K Garrison, S Harris, D Quinn; *National Clinical Research-Richmond, Inc, Richmond, VA:* J Scott, S Ayers, T Beasley, Finney, R Gordon, J Hoekstra, W Jeter, C Young; *Nebraska Heart Institute, Lincoln, NE:* P Dionisopoulos, C Godfrey, R Holcomb, S Krenk; *NJ Heart, Linden, NJ:* P Randhawa, S Agarwal, E Almond, E Capstraw, A Geraldo-Abache, S Kuchipudi, L Pasupuleti, C Sangiovanni, H Sheena, B Vargas; *North Alabama Research Center, LLC, Athens, AL:* E Hendrix, C Crews, J McNeese; *North Ohio Heart Center, Sandusky, OH:* M Traboulssi, A Bohn, K Humphrey, A Walton; *Northwest Heart Clinical Research, LLC, Arlington Heights, IL:* S Lupovitch, S Bellini, L Clemens, M Galindo, V Piszkiewicz, A Soni; *Norton Heart Specialists, Lousiville, KY:* J Lash, T Abell, V Flanery, J Hanrahan, D Mudd; *Novant Health Heart and Vascular Institute, Charlotte, NC:* J Pasquini, V Morton, J Nikitin, P Richards, C Sander, M Voelkers; *NYU Hudson Valley Cardiology, Cortlandt Manor, NY:* G Hamroff, L Bentivenga, K Fuerst-Carter, C Hametz, L Hollenweger, C Pankovic, A Solomon; *Ocala Research Institute, INC, Ocala, FL:* R Prashad, T Colacone,

M Green, P Lightcap, C McDonough, E Metivier, D Miller; *OhioHealth Research Institute, Mansfield, OH:* M Alton, D Grimwood-Fidler, G Heins, A Looney, L Orr, A Smith; *Oregon Health & Science University, Portland, OR:* W Clark, M Dolan, B Dugan, K Feest, J Foley; *Overlake Medical Clinics Cardiology, Bellevue, WA:* N Perlmutter, R Aviles, W Doucette, T Fortney, J Garceau, J Heywood, K Kanegae, C Kozlowski, D LeDoux, J Leggett, A Mahan, E McKinney, S Ostergard, J Smith, S Wagoner, S Yedinak, N Zilz; *Overlea Personal Physicians, Baltimore, MD:* B Kahn, A Campbell, V Coombs, J Phelps, E Sheridan, M Steinberg; *Palmetto Research Center, LLC, Spartanburg, SC:* R Littlefield, J Baty, A Clark, J Cooper, E Hames; *Parkview Research Center, Fort Wayne, IN:* W Collis, C Moeller, J Needham; *Pentucket Medical Associates, Haverhill, MA:* S Srivastava, S Bilazarian, C Ketis, K Roach; *Permian Research Foundation, Odessa, TX:* F Boccalandro, A Bryan; *Pottstown Medical Specialists, Inc, Pottstown, PA:* J Krantzler, N McClelland, T Muhlenberg, S Pickett; *Premier Healthcare, LLC, Bloomington, IN:* L Rink, E Anderson, A Brooks-Wolfe, B Litz, D Mobley; *Prevea Clinic, Inc., Green Bay, WI:* T Knutson, B Belanger, P Hermans, C Quinnell; *Primary Care Cardiology Research, Inc, Ayer, MA:* T Hack, E Fisher, L Morelli, S Sullivan; *PriMed Physicians, A Member of Northeast Medical Group, Yale New Haven Health, Trumbull, CT:* C Landau, D Ferguson, T Hilts; *Providence Saint Joseph Medical Center, Burbank, CA:* D Eisenberg, G Babar, M Fam, D Fernando, D Gallegos, G Kenegos, K Reed; *Regions Hospital-Heart Center, St. Paul, MN:* M Danish Rizvi, G Erie, C Eubanks, B Foster, J Kline, W Nelson; *Research Physicians Network Alliance, Hollywood, FL:* L Tami, M Abdur Rahman, J Viera Moreno; *Research Physicians Network Alliance, Pembroke Pines, FL:* P Krichmar, J Ferreira, D Marquez, H Sanchez-Lacayo, R Yunes; *Santa Rosa Cardiology Medical Group, Inc, Santa Rosa, CA:* J Hunter, E Battistelli, T Cook, R Iverson, M Suarez; *Saratoga Cardiology Assoc., PC/Saratoga Clinical Research, LLC, Saratoga Springs, NY:* D Kandath, S Frank, G Kostedt, J Nelson; *South Florida Research Group LLC, Miami, FL:* C Hamburg, L Diaz, E Hernandez, J Roberts, K Shatsky, E Torres; *South Oklahoma Heart Research, LLC, Oklahoma City, OK:* N Tahirkheli, T Adams, K Springer, W Springer; *Southwest Florida Research, LLC, Naples, FL:* J Talano, R Ficarra, L Leo, J Nolen, M Perez, G Rappley, N Szalanski; *Southwest Heart, Tucson, AZ:* B Peart, M Ford-Tarlton, K Peart, J Stephens; *St. Johns Center for Clinical Research, Ponte Vedra, FL:* D Schlager, E Schramm, M Rabalais, C Williamson; *St. Peter's Health Partners Medical Associates - Albany Associates in Cardiology, Albany, NY:* J DeSantis, K Benedetto, E Bursey, T Harting, R Muller, R Phang, E Roccario, P Schaummann-Boyle, A Zuchelkowski; *Tallahassee Research Institute Inc, Tallahassee, FL:* J Katopodis, K Gearld, P Knap, S Liebrich; *TCR Institute, LLC, Norwalk, CT:* I Lieber, L Ferree, F Stowe, M Sutton, D Wiseman; *Tenet Florida Physician Services, Jupiter, FL:* C Vogel, R Aggarwal, C Baroni, P Beck, J Blake, E Dagher, A Gryl, M Johnson, M Smith; *The Carl & Edyth Lindner Center for Research & Education at The Christ Hospital, Cincinnati, OH:* D Kereiakes, C DeFosse, J Schwartz; *The Center for Pharmaceutical Research, P.C., Kansas City, MO:* J Ervin, S Edwards, C Gorman, A Gorsuch, S Pomeroy; *The Polyclinic, Seattle, WA:* K Huehnergarth, K Davis, M Harder, M Lim, M Schrenker, S Yedinak; *The University of Iowa, College of Public Health, Preventive Intervention Center, Iowa City, IA:* J Robinson, J Cayler, M Cherrico, D Chun-Furlong, J De La Garza; *Trinity Medical Center, Rock Island, IL:* A Pothula, C Antonio-Drabek, A Bradley, R Buresh, T Hass, C Lopez*; UCH-MHS, Colorado Springs, CO:* J Strader, Jr., A Donlin, E Ensminger, H Garcia, A Gneiting, E Graf, D Greenberg; *University of Alabama Medical Center, Birmingham, AL:* W Rogers, P Arora, T Morgan, L Saag, S Thorington; *University of Maryland, Westminster, MD:* S Jerome, L Black, A Gupta; *University of Missouri Health System, Columbia, MO:* K Aggarwal, K Belew, V Burkhardt, S

Collins, S Holland Clasby, A Lau-Sieckman; *Upstate Cardiology, Greenville, SC:* J Cebe, E Calhoun, C Kissam, L Major; *Verde Valley Medical Center, Cottonwood, AZ:* S Butman, K Bescak, D Bescak, A Bigelow, T Brown, S Davidson; *Virginia Heart, Falls Church, VA:* T Haddad, T Alexander, J Jain, S McClain, T Myhera, D Overbeck, B Torre, L Wotorson; *Watson Clinic LLP, Lakeland, FL:* J Canto, C Corneal, B Donley, N McGowan, K Prisoc, M Sharrett; *Wenatchee Valley Hospital & Clinics, Wenatchee, WA:* S Kaster, J Akers, H Darlington, J Gault, J Horner, C Roozen; *Westlake Medical Research, Thousand Oaks, CA:* I Loh, R Anderson, T Call, J Esaki, P Patel, J Plocky, J Raymond, C Rideaux, L Sprafka; *Westside Center for Clinical Research, Jacksonville, FL:* M Stich, T Alexander, C Andres, C Brown, M Buda, S Ciuica, B Minker, S Perry; *York Hospital, York, PA:* K McCullum, B Doty, S Gates, K Hutcheson.

Supplemental tables and figures

Supplemental Table 1: Association of *ADCY9* rs1967309 genotype with components of major vascular events among placebo-allocated participants

Type of event and rs1967309 genotype	Placebo-allocated	Hazard ratio (95% CI)	P-value
Coronary death or myocardial infarction			
GG	217/3421 (6.3%)	reference	
AG	318/4636 (6.9%)	1.09 (0.92, 1.29)	p=0.42 (additive)
AA	103/1520 (6.8%)	1.08 (0.85, 1.36)	p=0.61 (genotypic)
Coronary revascularization			
GG	283/3421 (8.3%)	reference	
AG	340/4636 (7.3%)	0.89 (0.76, 1.04)	p=0.66 (additive)
AA	125/1520 (8.2%)	1.00 (0.81, 1.24)	p=0.25 (genotypic)
**MAJOR CORONARY EVENT**			
GG	397/3421 (11.6%)	reference	
AG	516/4636 (11.1%)	0.96 (0.84, 1.10)	p=0.93 (additive)
AA	180/1520 (11.8%)	1.03 (0.86, 1.23)	p=0.69 (genotypic)
Presumed ischaemic stroke			
GG	83/3421 (2.4%)	reference	
AG	113/4636 (2.4%)	1.02 (0.77, 1.35)	p=0.64 (additive)
AA	32/1520 (2.1%)	0.88 (0.58, 1.32)	p=0.75 (genotypic)
**MAJOR VASCULAR EVENT**			
GG	468/3421 (13.7%)	reference	
AG	615/4636 (13.3%)	0.97 (0.86, 1.10)	p=0.85 (additive)
AA	205/1520 (13.5%)	0.99 (0.84 ,1.17)	p=0.90 (genotypic)

Major coronary event is a composite endpoint including coronary death or myocardial infarction, or coronary revascularization. Major vascular event is a composite endpoint including major coronary event or presumed ischaemic stroke.

Supplemental Table 2: Effects of anacetrapib on blood pressure at trial midpoint, by *ADCY9* genotype

rs1967309 genotype	N	Mean (SE) (mmHg)		Absolute difference (mmHg)	P-value for interaction (anacetrapib x genotype)*
		Anacetrapib-allocated (n=8863)	Placebo-allocated (n=8747)		
**a) Systolic blood pressure**					
GG	6417	130.15 (0.31)	128.57 (0.31)	1.58	
AG	8398	129.51 (0.28)	129.37 (0.28)	0.13	p=0.47 (additive)
AA	2795	130.10 (0.47)	129.38 (0.46)	0.71	p=0.33 (genotypic)
Overall	17610	129.84 (0.19)	129.09 (0.19)	0.75	
**b) Diastolic blood pressure**					
GG	6417	77.19 (0.19)	76.54 (0.20)	0.65	
AG	8398	77.04 (0.17)	76.90 (0.17)	0.14	p=0.59 (additive)
AA	2795	77.42 (0.28)	76.76 (0.29)	0.66	p=0.32 (genotypic)
Overall	17610	77.16 (0.11)	76.75 (0.12)	0.41	

Unadjusted mean (SE: standard error) values are shown by randomized treatment and genotype among participants with blood pressure measurements available at both randomization and trial midpoint.

*P-values for interaction are based on a model for the blood pressure measurement at trial midpoint adjusted for 5 principal components of ancestry and the blood pressure measurement at randomization. P-values for interaction based on a model for the blood pressure measurement at trial midpoint adjusted for 5 principal components of ancestry only are as follows: systolic blood pressure p=0.10 (additive), p=0.06 (genotypic); diastolic blood pressure p=0.72 (additive), p=0.29 (genotypic).

Type of event and rs1967309 genotype	Anacetrapib-allocated	Placebo-allocated	Hazard ratio (95% CI)	P-value for interaction (anacetrapib x genotype)
**Coronary death or myocardial infarction**				
GG	201/3553 (5.7%)	217/3421 (6.3%)	0.89 (0.73-1.08)	
AG	266/4560 (5.8%)	318/4636 (6.9%)	0.85 (0.72-1.00)	p=0.40 (additive)
AA	80/1520 (5.3%)	103/1520 (6.8%)	0.76 (0.57-1.02)	p=0.68 (genotypic)
All participants	547/9633 (5.7%)	638/9577 (6.7%)	0.85 (0.75-0.95)	
**Coronary revascularization**				
GG	263/3553 (7.4%)	283/3421 (8.3%)	0.89 (0.75-1.05)	
AG	327/4560 (7.2%)	340/4636 (7.3%)	0.98 (0.84-1.14)	p=0.73 (additive)
AA	116/1520 (7.6%)	125/1520 (8.2%)	0.91 (0.70-1.17)	p=0.70 (genotypic)
All participants	706/9633 (7.3%)	748/9577 (7.8%)	0.93 (0.84-1.03)	
**MAJOR CORONARY EVENT**				
GG	372/3553 (10.5%)	397/3421 (11.6%)	0.90 (0.78-1.03)	
AG	475/4560 (10.4%)	516/4636 (11.1%)	0.93 (0.82-1.06)	p=0.87 (additive)
AA	158/1520 (10.4%)	180/1520 (11.8%)	0.86 (0.69-1.06)	p=0.78 (genotypic)
All participants	1005/9633 (10.4%)	1093/9577 (11.4%)	0.91 (0.83-0.99)	
**Presumed ischaemic stroke**				
GG	90/3553 (2.5%)	83/3421 (2.4%)	1.05 (0.78-1.41)	
AG	111/4560 (2.4%)	113/4636 (2.4%)	1.00 (0.77-1.30)	p=0.73 (additive)
AA	39/1520 (2.6%)	32/1520 (2.1%)	1.21 (0.76-1.93)	p=0.78 (genotypic)
All participants	240/9633 (2.5%)	228/9577 (2.4%)	1.05 (0.87-1.25)	
**MAJOR VASCULAR EVENT**				
**GG**	**451/3553 (12.7%)**	**468/3421 (13.7%)**	**0.92 (0.81-1.05)**	
**AG**	**572/4560 (12.5%)**	**615/4636 (13.3%)**	**0.94 (0.84-1.06)**	p=0.93 (additive)
**AA**	**193/1520 (12.7%)**	**205/1520 (13.5%)**	**0.93 (0.76-1.13)**	p=0.96 (genotypic)
**All participants**	**1216/9633 (12.6%)**	**1288/9577 (13.4%)**	**0.93 (0.86-1.01)**	

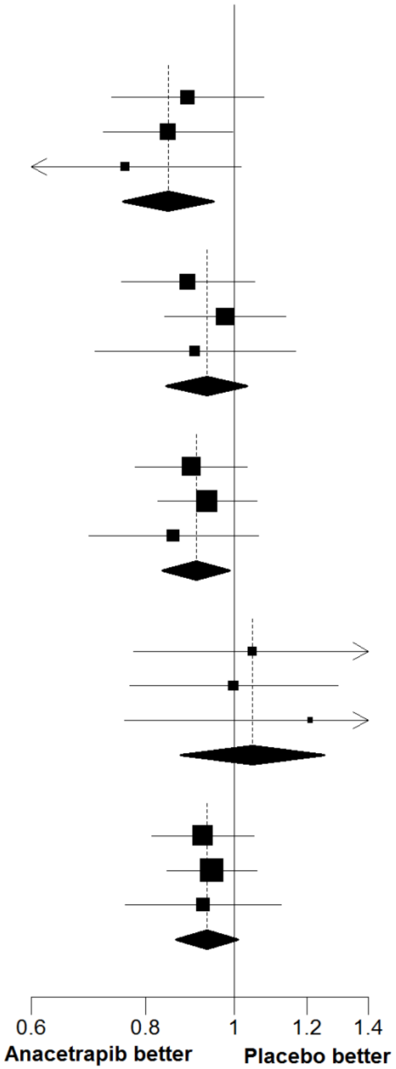

Supplemental Figure 1: Effects of anacetrapib on components of major vascular events, by *ADCY9* genotype

Major coronary event is a composite endpoint including coronary death or myocardial infarction, or coronary revascularization. Major vascular event is a composite endpoint including major coronary event or presumed ischaemic stroke.
